# Recent and Ongoing Horizontal Transfer of Mitochondrial Introns Between Two Fungal Tree Pathogens

**DOI:** 10.3389/fmicb.2021.656609

**Published:** 2021-06-02

**Authors:** Chase G. Mayers, Thomas C. Harrington, Alvan Wai, Georg Hausner

**Affiliations:** ^1^Department of Plant Pathology and Microbiology, Iowa State University, Ames, IA, United States; ^2^Department of Microbiology, University of Manitoba, Winnipeg, MB, Canada

**Keywords:** Rapid ‘Ōhi‘a Death, Rapid Ohia Death, mobile introns, horizontal gene transfer, forest pathology, Microascales, Ceratocystidaceae, *Ceratocystis*

## Abstract

Two recently introduced fungal plant pathogens (*Ceratocystis lukuohia* and *Ceratocystis huliohia*) are responsible for Rapid ‘ōhi‘a Death (ROD) in Hawai‘i. Despite being sexually incompatible, the two pathogens often co-occur in diseased ‘ōhi‘a sapwood, where genetic interaction is possible. We sequenced and annotated 33 mitochondrial genomes of the two pathogens and related species, and investigated 35 total *Ceratocystis* mitogenomes. Ten mtDNA regions [one group I intron, seven group II introns, and two autonomous homing endonuclease (HE) genes] were heterogeneously present in *C. lukuohia* mitogenomes, which were otherwise identical. Molecular surveys with specific primers showed that the 10 regions had uneven geographic distribution amongst populations of *C. lukuohia*. Conversely, identical orthologs of each region were present in every studied isolate of *C. huliohia* regardless of geographical origin. Close relatives of *C. lukuohia* lacked or, rarely, had few and dissimilar orthologs of the 10 regions, whereas most relatives of *C. huliohia* had identical or nearly identical orthologs. Each region included or worked in tandem with HE genes or reverse transcriptase/maturases that could facilitate interspecific horizontal transfers from intron-minus to intron-plus alleles. These results suggest that the 10 regions originated in *C. huliohia* and are actively moving to populations of *C. lukuohia*, perhaps through transient cytoplasmic contact of hyphal tips (anastomosis) in the wound surface of ‘ōhi‘a trees. Such contact would allow for the transfer of mitochondria followed by mitochondrial fusion or cytoplasmic exchange of intron intermediaries, which suggests that further genomic interaction may also exist between the two pathogens.

## Introduction

Mobile introns and homing endonuclease (HE) genes are diversity-generating elements that contribute to the size and diversity of mitochondrial genomes in fungi and can potentially facilitate horizontal gene transfer between fungal species. Mitochondrial introns can be classified based on splicing mechanism, structure, and intron encoded proteins (IEPs) into either “group I” or “group II,” both of which can be transferred either vertically or horizontally (Belfort et al., 2002; Lang et al., 2007; Hausner, 2012; McNeil et al., 2016; Zubaer et al., 2018). Group I introns self-splice from precursor RNA with the help of their own IEPs and/or nuclear-encoded proteins (Lambowitz and Perlman, 1990; Cech et al., 1994; Wallweber et al., 1997; Vicens et al., 2008) to become linear RNA intermediates (Cech, 1990). Mobile group I introns use intron encoded HEs to recognize large (∼20–30 bp) target sites in DNA (Chevalier and Stoddard, 2001) and invade intron-negative alleles (“intron homing”) (Dujon, 1989; Belfort and Perlman, 1995; Stoddard, 2011). Some group II introns can self-splice and catalyze their mobility with intron-encoded HEs (Mullineux et al., 2010), but most group II introns require intron-encoded reverse transcriptase/maturases and host-encoded factors to efficiently splice, which may result in the formation of branched or lariat RNA intermediates. The lariat RNA can form a complex with its IEP, and the ribonucleoprotein complex can invade specific sites in intron-negative alleles (“retrohoming”) (Gray, 1998; Rosewich and Kistler, 2000; Matsuura et al., 2001; Lambowitz and Zimmerly, 2011; McNeil et al., 2016). In addition to conferring mobility to group I, or in some instances to group II introns, intron encoded homing endonuclease genes (HEGs) can move independently of their intron partners, and such autonomous HEGs can catalyze and direct their own homing mobility to HEG-minus target sites (Sellem and Belcour, 1997; Belfort et al., 2002; Toor and Zimmerly, 2002; Stoddard, 2011; Hafez and Hausner, 2012; Megarioti and Kouvelis, 2020).

Rapid ‘ōhi‘a Death (ROD) is a new, devastating disease on Hawai‘i Island and Kaua’i Island of Hawai‘i (Keith et al., 2015; Mortenson et al., 2016) causing dramatic mortality of the ecologically and culturally important native tree ‘ōhi‘a lehua (*Metrosideros polymorpha*). Mortality is associated with two species of the fungal genus *Ceratocystis* (Ascomycota: Microascales: Ceratocystidaceae), *Ceratocystis lukuohia* and *Ceratocystis huliohia* (Barnes et al., 2018), whose spores are likely spread in windborne frass of wood-boring ambrosia beetles (Roy et al., 2019, 2020). Neither *Ceratocystis* species is considered native to Hawai‘i and they were likely separately introduced on nursery stock. The two ‘ōhi‘a pathogens are not sexually compatible (Barnes et al., 2018) and exist in two different geographic clades of *Ceratocystis*: *C. lukuohia* in the “Latin American Clade” (LAC) with many other aggressive pathogens (Engelbrecht and Harrington, 2005; Li et al., 2017), and *C. huliohia* in the “Asian Australian Clade” (AAC) with generally less aggressive pathogens (Thorpe et al., 2005; Li et al., 2017). *C. lukuohia* causes staining and death of the ray parenchyma of ‘ōhi‘a sapwood (Hughes et al., 2020) and is the major cause of mortality, whereas *C. huliohia* causes canker-stain symptoms, branch death, and only occasionally death of the whole tree (Barnes et al., 2018). Horizontal gene transfer has enabled host range expansion in other fungal pathogens (Mehrabi et al., 2011) and could explain why both species are aggressive pathogens on ‘ōhi‘a. The two pathogens infect wounds and often co-colonize diseased ‘ōhi‘a sapwood where genetic exchange through hyphal anastomosis, that is, the fusion of hyphal tips or germlings (Fitzpatrick, 2012; Cheeseman et al., 2014; Feurtey and Stukenbrock, 2018) is hypothetically possible. We sought to find evidence of such horizontal exchange by comparing mitochondrial genomes of the pathogens from across Hawai‘i Island and of *Ceratocystis* relatives in the LAC and AAC.

## Materials and Methods

### Isolates and DNA Extraction

Cultures of *C. huliohia* and *C. lukuohia* were obtained from recently killed ‘ōhi‘a trees across Hawai‘i Island, in most cases by baiting from stained sapwood tissue with slices of carrot root and transferring from ascospore masses that formed on the tips of perithecia (Thorpe et al., 2005; Barnes et al., 2018). Among the 89 studied isolates of *C. lukuohia*, 15 were selected from across the island for genome sequencing, including several isolates collected in 2014–2018 from the believed origin of the epidemic in Lower Puna. The genomes of three geographically scattered isolates of *C. huliohia* were also sequenced, as well as the genomes of additional representatives of the LAC and AAC of *Ceratocystis* in the collection at Iowa State University (Baker et al., 2003; Engelbrecht and Harrington, 2005; Johnson et al., 2005; Thorpe et al., 2005; Li et al., 2017; Liu et al., 2018). Isolates were stored in 15% glycerol at −80°C. The identity of each isolate was confirmed by ITS-rDNA sequencing.

Isolates were grown on malt yeast extract agar (MYEA: 2% malt extract, Difco; 0.2% yeast extract, Difco; 1.5% agar) for 5–7 days at room temperature and light. Surface growth, which consisted mostly of hyphae and conidiophores with cylindrical conidia, was scraped with a sterile spatula, and DNA was extracted using the ProMega Wizard ^ ®^ Genomic DNA Purification Kit (Promega, Madison, WI, United States) and the “Plant Tissue” protocol with minor modifications (maceration of fresh tissue with 1 mm glass beads rather than liquid nitrogen and grinding). DNA concentration was quantified with a Qubit 2.0 fluorometer (Invitrogen, Carlsbad, CA, United States).

### Genome Sequencing

Illumina MiSeq reads (2 × 300 bp, paired ends, 600-cycle, v3 reagent kit) were generated by the Iowa State University DNA Facility for 33 isolates of *Ceratocystis* (Table 1). The genomes were produced in three batches, and each batch was run on one or two flow cells with separate indexes for each isolate. Adapter sequences were removed and raw reads were quality trimmed using BBDuk 1.0 as implemented in Geneious v. 11 (Biomatters Ltd., Auckland, New Zealand) with minimum quality 13 from both ends. The paired end reads of each isolate were merged into a “trimmed read pool” before assembly.

**TABLE 1:** Mitogenome size, introns, and estimated ratio of mitochondrial to nuclear genome coverage of *Ceratocystis* isolates.

**Clade**	**Species**	**Location**	**Isolate**	**GenBank accession**	**Mean coverage**	**mt:nuc ratio^a^**	**Entire mt genome size**	**Intron count**	**Total size of introns (bp)**	**% Introns**	**mt genome size, no introns (bp)**
AAC^b^	*C. changhui*	China	C3371 (CBS 139742)	MT331818	1,047×	46	177,592	70	127,368	71.72	50,224
	*C. huliohia*	Hawai‘i	C4189	MT331821	1,065×	42	155,136	62	107,598	69.36	47,538
	*C. huliohia*	Hawai‘i	C4194	MT331822	774×	35	155,137	62	107,598	69.36	47,539
	*C. huliohia*	Hawai‘i	C4381	MT331820	1,228×	51	155,137	62	107,598	69.36	47,539
	*C. polychroma*	Indonesia	C2240 (CBS 115775)	MT331817	601×	68	161,224	67	114,494	71.02	46,730
	*C. uchidae*	Hawai‘i	C1714 (CBS 115164)	MT331819	1,106×	32	159,132	65	111,577	70.12	47,555
LAC^c^	*C. cacaofunesta*	Brazil	C1593	JX185564^d^	N/A^d^	N/A^d^	103,147	38	58,547	56.76	44,600
	*C. cacaofunesta*	Brazil	C1983 (CBS 115172)	MT331846	373×	15	107,390	42	62,980	58.65	44,410
	*C. colombiana*	Colombia	C1945	MT331845	1,296×	39	131,365	52	84,277	64.15	47,088
	*C. fimbriata* ex *Coffea*	Costa Rica	C1551	MT331842	1,001×	78	116,877	45	69,381	59.36	47,496
	*C. fimbriata* ex *Eucalyptus*	Brazil	C1442 (CBS 115174)	MT331848	508×	21	96,592	33	52,195	54.04	44,397
	*C. fimbriata* ex *Hevea*	Guatemala	C1944	MT331841	14×	N/A^*e*^	133,368	53	86,286	64.70	47,082
	*C. fimbriata* ex *Ipomoea*	N. Carolina	C1421 (CBS 114723)	MT331847	547×	27	145,765	57	96,920	66.49	48,845
	*C. fimbriata* ex *Mangifera*	Brazil	C1688 (CBS 114721)	MT331849	503×	17	97,079	35	52,685	54.27	44,394
	*C. fimbriata* ex *Spathodea*	Cuba	C1811	MT331843	920×	19	115,345	44	69,435	60.20	45,910
	*C. fimbriata* ex *Syngonium*	Florida	C1809 (CBS 115167)	MT331840	676×	38	149,807	60	99,464	66.39	50,343
	*C. fimbriata* ex *Syngonium*	Hawai‘i	C4121	MT331839	969×	58	148,618	59	98,276	66.13	50,342
	*C. fimbriata* ex *Xanthosoma*	Costa Rica	C1780 (CBS 115165)	MT331838	367×	20	144,128	58	93,741	65.04	50,387
	*C. lukuohia*	Hawai‘i	C4123	MT331834	1,198×	57	130,154	51	79,874	61.37	50,280
	*C. lukuohia*	Hawai‘i	C4124	MT331823	765×	35	144,435	56	94,131	65.17	50,304
	*C. lukuohia*	Hawai‘i	C4128	MT331828	1,321×	48	132,741	52	82,461	62.12	50,280
	*C. lukuohia*	Hawai‘i	C4183	MT331829	1,459×	32	132,742	52	82,461	62.12	50,281
	*C. lukuohia*	Hawai‘i	C4185	MT331826	807×	41	133,859	53	83,577	62.44	50,282
	*C. lukuohia*	Hawai‘i	C4187	MT331833	898×	38	130,153	51	79,874	61.37	50,279
	*C. lukuohia*	Hawai‘i	C4212 (CBS 142792)	MT331837	592×	23	127,076	50	76,796	60.43	50,280
	*C. lukuohia*	Hawai‘i	C4253	MT331835	1,014×	47	130,154	51	79,874	61.37	50,280
	*C. lukuohia*	Hawai‘i	C4321	MT331824	684×	35	143,252	56	92,972	64.90	50,280
	*C. lukuohia*	Hawai‘i	C4341	MT331830	919×	32	131,236	52	80,956	61.69	50,280
	*C. lukuohia*	Hawai‘i	C4342	MT331825	888×	34	141,540	56	91,260	64.48	50,280
	*C. lukuohia*	Hawai‘i	C4364	MT331827	1,351×	61	133,825	53	83,543	62.43	50,282
	*C. lukuohia*	Hawai‘i	C4370	MT331831	1,012×	64	131,236	52	80,956	61.69	50,280
	*C. lukuohia*	Hawai‘i	C4372	MT331836	1,424×	70	127,077	50	76,796	60.43	50,281
	*C. lukuohia*	Hawai‘i	C4379	MT331832	978×	51	131,236	52	80,956	61.69	50,280
	*C. papillata*	Colombia	C1750	MT331844	199×	N/A^*e*^	121,288	49	77,018	63.50	44,270
	*C. platani*	Italy		LBBL01000003^d^	N/A^d^	N/A^d^	116,095	44	66,975	57.69	49,120

*^*a*^Ratio of coverage of the mitochondrial contig to first coverage of the largest nuclear contig. ^*b*^Asian-Australian Clade. ^*c*^Latin American Clade. ^*d*^Sequence not generated in this study. ^*e*^Unable to estimate coverage of the nuclear contigs due to contamination of bacterial DNA sequences.*

Because the original intention of this project was to study nuclear genomes, the initial *de novo* assemblies used a complex workflow in Geneious that separated the assembly of high- and low-copy reads to save computing power on consumer machines. This workflow (Data Sheet 1) is the method by which all mitogenomes were originally assembled. However, later simpler assemblies using Geneious *de novo* assembly with default settings (from a subsample of the read pool when necessary) or NOVOPlasty (Dierckxsens et al., 2017) produced identical mitochondrial contigs for all isolates.

Cultures of two isolates (C1750 and C1944) had bacterial contaminants (*Paenibacillus* spp.) that became apparent when examining their assembled contigs. To rectify this, the original trimmed read pools for the two isolates were first assembled to a circularized genome (GenBank CP018620) and plasmid (GenBank CP018621) of *Paenibacillus xylanexedans* (the most closely related genome available per NCBI BLAST) as well as the circularized genome (GenBank CP022655) of another close relative, *Paenibacillus* sp. RUD330. Only the reads that did not assemble to these bacterial contigs were used for genome assembly as described above.

### Recovery of Mitochondrial Genomes

For all isolates, the largest contig of the high coverage assembly was the mitochondrial genome. This contig was automatically circularized in all isolates except C1944 and C4124, for which failure to circularize was due to erroneous assemblies of low-quality reads at the ends of the contigs, and the contigs were manually circularized. Two additional *Ceratocystis* mitochondrial genomes were available on GenBank: *Ceratocystis cacaofunesta* JX185564 (Ambrosio et al., 2013) and *Ceratocystis platani* LBBL01000003.1 (Belbahri, 2015, unpublished). Circular mitochondrial genomes were reverse-complemented if necessary and their origins set to match the annotated JX185564 genome, that is, at the beginning of the conserved sequence “GTGA” 21 bp upstream from the 5′ end of the *rnl* rRNA gene.

### Alignment and Annotation of Mitochondrial Genomes

The set of 35 circularized mitochondrial genomes were manually aligned in Geneious for a total alignment length of 225,988 bp. Preliminary annotations were created using JX185564 as a reference and the “Transfer Annotations” function in Geneious, but many gene and intron annotations required adjustments. Mitochondrial rDNA annotations were adjusted by comparing to the annotated mitochondrial genomes of another member of the Ceratocystidaceae, *Endoconidiophora resinifera* (Zubaer et al., 2018). Intron boundaries were adjusted so that group I introns ended with terminal 3′ omega-Gs, and group II introns generally began with a 5′ GUGYG and ended with a 3′ AY (Hausner, 2012; Hausner et al., 2014). Each putative gene sequence was checked via NCBI blastp for homology with known proteins in the NCBI protein database (pdb) to eliminate discrepancies in gene length, intron placement, and incorrect start/stop codons. Apparent introns and tRNAs in the alignment were characterized with the online tool RNAweasel^1^ (Lang et al., 2007) and applied to the mitochondrial alignment in Geneious. Intron classifications, including those not recognized by RNAweasel, were augmented by comparing their secondary structure to known intron classes (Zubaer et al., 2018). For the 10 regions of interest, putative IEPs were determined via either blastx search of the NCBI non-redundant protein sequences database (nr) or NCBI protein database (pdb). Introns were numbered according to their order (from 5′ to 3′) within each gene in our alignment (e.g., *cox1* i1, *cox1* i2 … *nad2* i1, *nad2* i2 …), without regard to how the introns were numbered in previous studies.

To compare intron frequency and diversity across all *Ceratocystis* isolates, each set of homologous introns was separately extracted and aligned with Geneious Alignment (global, 93% similarity, gap open penalty 12, gap extension penalty 3, and refinement iterations 2), and the resulting distance matrix (set to % identity) was exported.

### Detection of Mitochondrial Variants Among Isolates

Initially, the mitochondrial genomes for *C. lukuohia* isolates C4128, C4183, and C4185 were aligned using the built-in “Geneious Alignment” function (Global alignment, free end gaps, 65% similarity, gap open penalty 30, gap extension penalty 3). Variation among the three genomes suggested three putative mobile group II introns: “SPAM1,” “SPAM2,” and “SPAM3” (SPecies A Mitochondrial elements, after “Species A,” the previous informal name for *C. lukuohia*). Additional mitochondrial variants (SPAMs) among the 15 *C. lukuohia* genomes were later discovered in the final manually aligned set of all mitochondrial genomes and were numbered in order of discovery.

### Phylogenetic Analyses

Three alignments were created for Bayesian analyses. The first, a general mitochondrial alignment, was assembled using exons of 15 mitochondrial genes: *rnl* rRNA; *rps3*; *nad2*; *nad3*; *cox2*; *nad4L*; *nad5*; *cox1*; *nad1*; *nad4*; *atp8*; *atp6*; *rns* rRNA; *cox3*; and *nad6.* Sequences of *cob* were excluded because of the co-conversion of a flanking exon by one of the heterogeneously present introns, SPAM9, which caused aberrant phylogenetic placement of SPAM9-positive isolates. Sequences of *atp9* were also excluded, because premature stop codons in nearly all isolates suggested they may be nonfunctional and therefore degenerated, as discussed further in the results section. The mitochondrial exon alignment had 17,588 characters, of which 45 were variable^2^. The second alignment included only the DNA sequences of *cob* exons to illustrate the aberrant placement of SPAM9-positive isolates. The *cob*-only alignment had 1,179 characters, of which 25 were variable^3^. The third alignment, a nuclear phylogeny for comparison with the mitochondrial phylogenies, was produced by extracting the mating type genes *MAT1-2-1* and *MAT1-1-2* from each nuclear assembly. Introns were excluded for analysis, which left 2628 aligned characters, 650 of which were variable^4^. Where multiple isolates had identical sequences, only one representative sequence was used for analysis, and then the isolates with redundant sequences were added in the tree illustrations. All three alignments were partitioned by gene and further by all three codon positions in protein-coding genes. Models for each partition were selected using PartitionFinder 2 (Lanfear et al., 2017) with AICc (converted Akaike Information Criterion) and a greedy algorithm (Lanfear et al., 2012) powered by PhyML (Guindon et al., 2010).

Bayesian phylogenetic trees were produced from all alignments using MrBayes v3.2.2 ×64 (Ronquist et al., 2012). Each analysis was run with the models suggested by PartitionFinder 2 for a number of generations sufficient to achieve a standard deviation of split frequencies less than 0.01 (nuclear mating genes, 1,000,000 generations; *cob* only, 4,000,000; multigene mitochondrial genes, 5,000,000) with default settings used otherwise. A consensus tree was generated (sumt) with a burnin value of 15% and visualized in FigTree 1.4.0. The unrooted mitochondrial trees were manually rearranged in FigTree to visually divide the AAC and LAC, and the nuclear gene tree was rooted to the outgroup *Ceratocystis variospora* isolate C1963 (a member of the North American Clade).

Separate alignments were produced for analyses of the 10 SPAM regions and their orthologs: SPAM1 (300 variable/2621 total characters with gaps treated as missing data), SPAM2 (166/2602), SPAM3 (0/1082; all sequences identical), SPAM4 (52/2759), SPAM5 (572/2492), SPAM6 (32/2740), SPAM7 (2/3092), SPAM8 (26/1154), SPAM9 (41/1368), and SPAM10 (9/1197). Within each alignment, the relative sequence similarity was generally too small for Bayesian analysis, so a separate UPGMA tree (Tamura-Nei distance model) was generated for every SPAM alignment using Geneious Tree Builder.

### Development of Markers for PCR-Based Intron Surveys

**FIGURE 1:**
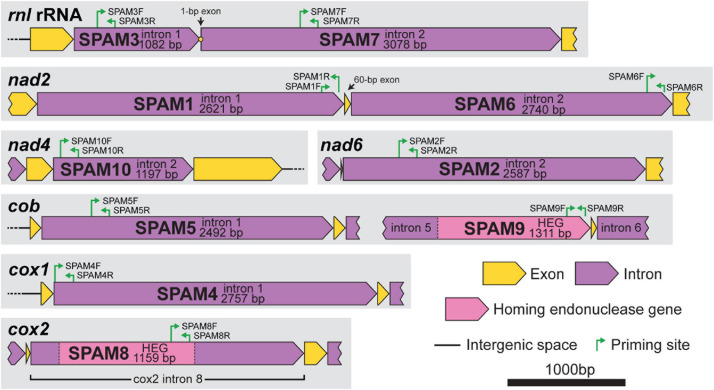
Gene context and map of standard primers (internal primers used for PCR surveys) for each of the 10 heterogenous mitochondrial elements (SPAM1–SPAM10) in *Ceratocystis lukuohia.* Primer sequences are detailed in Table 2. All regions are to scale; bar = 1,000 bp.

The absence or presence of SPAMs in the total population of 89 *C. lukuohia* isolates and 17 *C. huliohia* isolates was determined through PCR surveys using unique primer pairs for each of the 10 SPAM regions (Figure 1 and Table 2). Primers were designed in Geneious (modified version of Primer3 2.3.7) using a target Tm 60°C and product size 100–400 bp (but as close to 150 bp as possible). Template DNA was extracted as described earlier and adjusted to 1 ng/μL. PCR reaction mixtures were composed of 9.125 μL H_2_O, 5 μL 5× Promega GoTaq ^®^ Green Flexi Reaction Buffer, 2.5 μL 2 mM dNTPs, 4 μL 25 mM MgCl_2_, 1.25 μL DMSO, 0.5 μL of both forward and reverse primers at 50 mM, 0.125 μL ProMega GoTaq ^®^ Flexi DNA Polymerase, and 2 μL DNA template, for a total reaction volume of 25 μL. Cycling conditions were 95°C for 4 min; 30 cycles of 95°C for 1 min, 55°C for 1 min, and 72°C for 1 min; 72°C for 9 min; and 4°C hold. The PCR products, including appropriate positive (from an isolate known to have that SPAM) and negative (water) controls, were run on 1.8% agarose gels and visualized after ethidium bromide staining (15 m in 1 L 0.5 μg/mL EtBr, 2 m destain in 1 L water) with a Gel Doc XR+ (Bio-Rad, Hercules, CA, United States). Each of the 10 primer pairs specific to the 10 putatively mobile regions (SPAMs) amplified strong products with the DNA extracted from each of the 17 tested isolates of *C. huliohia*. When the primers were used on *C. lukuohia* template, a strong band was interpreted as a positive for the presence of the intron in that isolate; the absence of bands was interpreted as the absence of the intron in that isolate; and a weak band was interpreted as the putative absence of the intron in that isolate. Weak bands may have been the result of alternative, but less efficient (with some base mismatches) priming sites elsewhere in the genome (most likely within unrelated introns), and weak bands often had different product sizes or multiple products in contrast to a strong single band. In addition to the “standard” primers (both primers internal to the SPAM), additional special-use primer pairs were designed for certain SPAMs. “Anchor” primer pairs, which have one primer within the intron and the other just outside the intron, were used to confirm SPAM insertion sites because they only produce product if the SPAM is inserted in the same position. “Spanning” primer pairs (primers on both sides of the intron insertion point) were used to confirm negative results, because they only produce product if the SPAM is absent; the large SPAM elements, when present, cause the priming sites to be too distant for amplification.

**TABLE 2:** Primers used for PCR detection of mobile mitochondrial elements (SPAMs).

**Targeted intron**	**Primer name**	**Sequence**	**Product size**
SPAM1	SPAM1F	ACGATCTCTTACACCGGGGA	144 bp
	SPAM1R	TCCCATCATCAGCAGCCTTG	
SPAM2	SPAM2F	ACGGAGCCCCCATAGTAGTT	147 bp
	SPAM2R	AGTACCGTCCCTCTCGTACC	
SPAM3	SPAM3F	CTTCGGGCTCTGAGTCCAGA	147 bp
	SPAM3R	CAGGGCTTACCATGTTCCGT	
SPAM4	SPAM4F	TGCGCCGTGTTTGTAAATGT	150 bp
	SPAM4R	GCAACGATAGCCTTGAACGG	
SPAM5	SPAM5F	CGGGAAGTAACCGCCTATGT	151 bp
	SPAM5R	TCACTTGACTTCGCCGGTAA	
SPAM6	SPAM6F	GCTACGAAGAGTACCAGGACG	140 bp
	SPAM6R	AACCCAGCGAGATTGTTACCA	
SPAM7	SPAM7F	GTCCCTGAATGTTTGCGCTT	150 bp
	SPAM7R	TACCCGATTGGCGTTCTGG	
SPAM8	SPAM8F	AAAGGAATCCAATGATAATGAGTACGG	149 bp
	SPAM8R	GGGACCTATTCTTTTAGAAGCTCTG	
SPAM9	SPAM9F	TCGCTGGCTATTGAGAAGCA	150 bp
	SPAM9R	ATTCTTTTAAATATCCGCCCAAAGG	
SPAM10	SPAM10F	AGGAAACTGTTTGCTATTATTAGCTGT	150 bp
	SPAM10R	TGTATCTAGCGTATGGCCTCTG	
SPAM11	SPAM11F	TATGATGAGTGGTATGGGCACG	150 bp
	SPAM11R	TCAGGAGTACCATCCCAGCT	
SPAM12	SPAM12F	TCGCAGACTTAACAAAGGTGAAA	150 bp
	SPAM12R	CGCCTTTCAACGCATACGG	

### Geographic Map

In order to display the geographic origin of collected isolates, a map of Hawai‘i Island was rendered in Maperitive v2.4.3. Heightmap data for terrain and shading was obtained from the NASA Shuttle Radar Topography Mission Global 3-arc-second (SRTMGL3) dataset downloaded from EOSDIS Earthdata^5^ and manually imported into Maperitive; other map information is derived from OpenStreetMap as implemented in Maperitive.

## Results

### Mitochondrial Genomes

**FIGURE 2:**
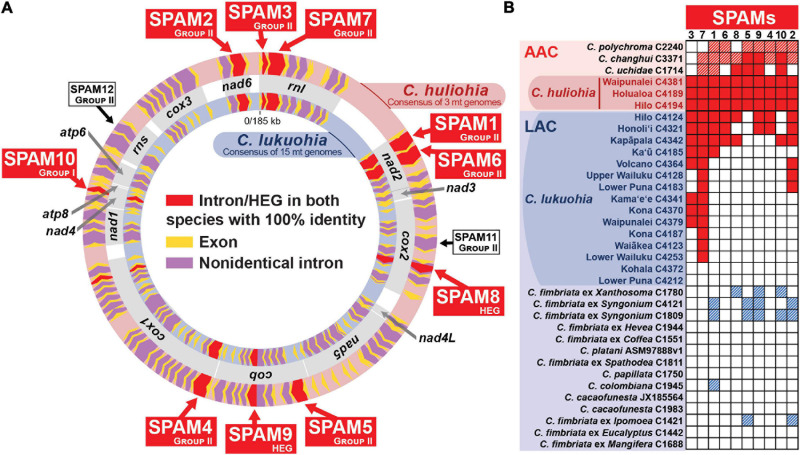
Ten “SPAM” (SPecies A Mitochondrial) elements heterogenous among *Ceratocystis lukuohia* genomes but identical and always present in *Ceratocystis huliohia*. **(A)** Mitochondrial genome maps for *C. lukuohia* and *C. huliohia*, with SPAMs putatively transferred from the Asian *C. huliohia* (outer circle) to the Latin American *C. lukuohia* (inner circle) indicated as red chevrons and named with red boxes. The red-shaded c*ox1* i17 (near the end of *cox1*) was found with 100% identity in both species but was also present with 100% identity in nearly all the studied genomes. White boxes indicate two putative SPAMs that were not found in studied *C. lukuohia* isolates. Mitochondrial genes are indicated by gray boxes between the genome maps and named in bold. **(B)** The presence and absence of SPAM mobile elements in mitogenomes of *C. lukuohia*, *C. huliohia* and other *Ceratocystis* species. Solid red boxes indicate present orthologs with 100% identity to the region in *C. huliohia*; red hashed boxes indicate an Asian-Australian Clade ortholog with less than 100% identity; blue hashed boxes indicate a Latin American Clade ortholog with less than 100% identity; white boxes indicate absence.

**FIGURE 3:**
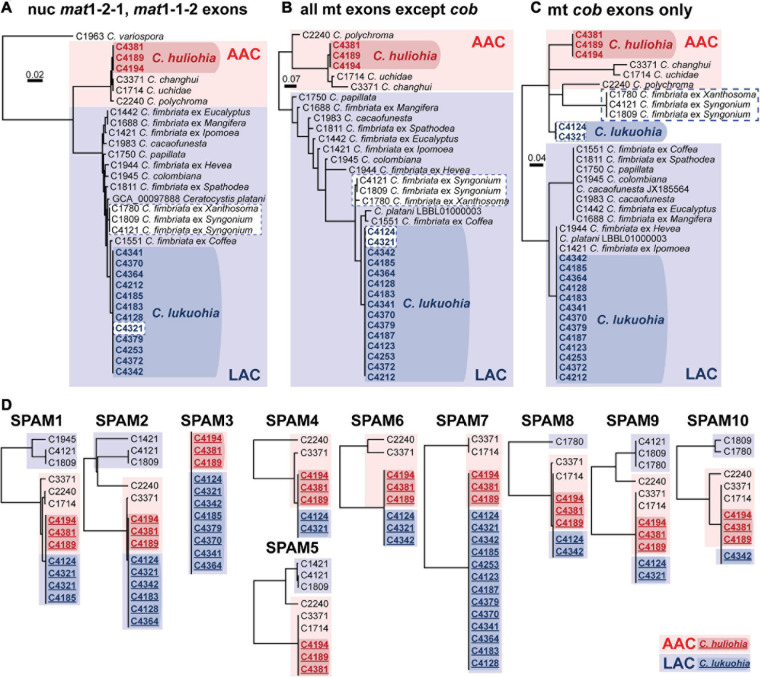
**(A–C)** Bayesian inference trees of exons of nuclear and mitochondrial genes of Latin American Clade (LAC) and Asian Australian Clade (AAC) species of *Ceratocystis*. **(A)** Nuclear genes (*mat1-2-1* and *mat1-1-2* exons). **(B)** All mitochondrial exons besides *cob*. **(C)** Exons of *cob*. **(D)** Separate UPGMA distance trees for each SPAM element. Members of the Asian-Australian Clade are in red, and those of the Latin American Clade are in blue. The SPAM9-positive LAC taxa are highlighted in white boxes with dashed blue borders in panel **(A–C)**, highlighting their aberrant phylogenetic placement due to co-conversion of *cob* by SPAM9. In D, sequences from *C. lukuohia* (bold blue, underlined, in dark blue box) and *C. huliohia* (bold red, underlined, in dark red box) are identical and group with other AAC species (plain text, in light red boxes) and apart from LAC sequences (plain text, in light blue boxes). Sequences of SPAM5 were not available for *C. lukuohia*.

We generated 33 mitochondrial genomes of various *Ceratocystis* species that ranged in size from 96,592 to 177,592 bp, generally in proportion to the number of introns they contained (Table 1). The genomes all had the same number of genes in the same order as other Ceratocystidaceae (Ambrosio et al., 2013; Zubaer et al., 2018) and nearly all Sordariomycetes (Aguileta et al., 2014) (Figure 2A) and each had the same 31 tRNA genes. Putative translations of all genes appeared functional except *atp9*, which included a premature stop codon in the middle of its CDS and whose function is presumably replaced by a nuclear-encoded homolog (Zubaer et al., 2018, 2021). The mitochondrial genomes were annotated and submitted to GenBank (Table 1), and we also produced new annotations for two genomes already available on GenBank, *C. platani* LBBL01000003.1 and *C. cacaofunesta* JX185564. We detected 90 introns (79 group I, 11 group II) among the 35 aligned *Ceratocystis* mitochondrial genomes (Supplementary Table 1). The phylogenies of nuclear exons (mating type genes) and mitochondrial exons each sorted all isolates into either the AAC or LAC group, with *C. huliohia* in the former and *C. lukuohia* in the latter, as expected (Figures 3A,B). Some mitochondrial introns were unique to the LAC or AAC, but most appeared to be orthologs present (but divergent) in both LAC and AAC species, suggesting shared histories of vertical descent (Supplementary Table 1).

### Heterogeneous Introns/HEGs in *Ceratocystis lukuohia* Are All Shared With *Ceratocystis huliohia*

The three *C. huliohia* mitogenomes were practically clonal (differing by only a single nucleotide), including completely identical introns (Table 1). In contrast, the 15 *C. lukuohia* mitogenomes varied significantly in size, from 127,076 to 144,435 bp (Table 1). The contribution of non-intronic regions to this diversity was negligible; intergenic differences consisted only of minor variations in single-nucleotide repeat length, a single-bp transversion in isolate C4370, and a 23-bp insert between *rnl* rRNA and *nad2* in one isolate, whereas exonic differences were found only in a single exon of *cob* in two isolates (C4124 and C4321), which caused aberrant phylogenetic placement (Figure 3C), as discussed below. The remaining and vast majority of mitogenome diversity in *C. lukuohia* was due to the absence or presence of nine specific regions. These nine variable sites in *C. lukuohia* were collectively termed “SPAMs” (SPecies A Mitochondrial regions) (Figures 2A,B). The other 57 introns detected in *C. lukuohia* were identical in their presence, sequence, and placement.

Each *C. lukuohia* SPAM had an identical ortholog in the exact same position in all three genomes of *C. huliohia* (Figure 2A). In sharp contrast, all of the 36 other orthologous introns shared by *C. lukuohia* and *C. huliohia* (except *cox1* i17, which was nearly identical in all species) differed at least slightly in sequence, as would be expected for vertically descendant orthologs (Supplementary Table 1). There were also identical orthologs of some SPAMs in the AAC species *Ceratocystis changhui* and *Ceratocystis uchidae*, but otherwise, SPAM orthologs were only found with reduced identity in all 3 AAC relatives and 5 out of 14 LAC relatives (Figure 2B and Supplementary Table 1). UPGMA analyses of SPAM orthologs grouped the *C. lukuohia* sequences with those of *C. huliohia* and sister to orthologs from AAC species rather than those of LAC species (Figure 3D), suggesting that the SPAMs in *C. lukuohia* are likely AAC-derived mobile DNA regions transferred from *C. huliohia* rather than ancestral LAC regions.

**FIGURE 4:**
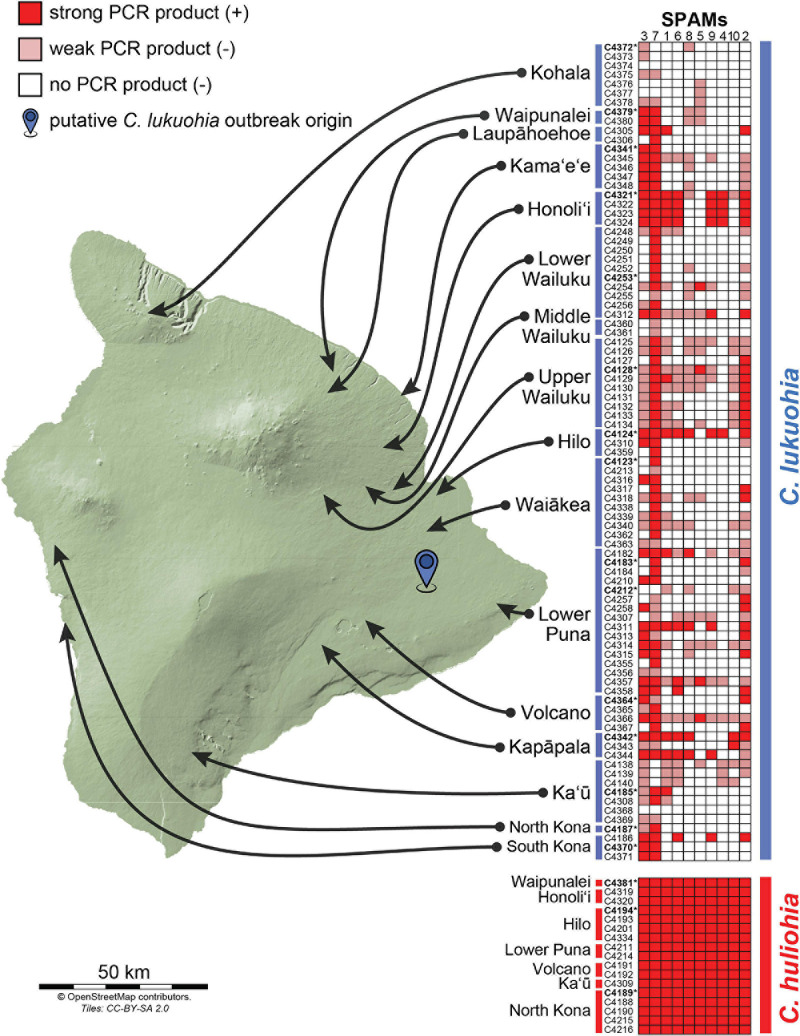
Results of PCR surveys for the 10 SPAM elements among 89 isolates of *C. lukuohia* and 17 isolates of *C. huliohia* across Hawai‘i Island using the primers in Table 2. Dark red boxes represent strong PCR bands (positive), faint red boxes represent weak or very weak PCR bands (interpreted as negative), and white boxes represent the absence of PCR bands (negative). Isolates for which genomes were sequenced in this study are in bold and followed by asterisks.

Because most SPAMs appeared to be Group II introns, and we noticed three group II introns in the *C. huliohia* mitogenomes that were absent in the 15 sequenced mitogenomes of *C. lukuohia* (Figure 2A), we designed PCR primers for each of these three group II introns (Table 2). One of them, *cob* i1, was quickly confirmed in four of the 89 isolates from across Hawai‘i Island before all genomic SPAMs had been characterized, and was called SPAM5 (Figure 4). Primer surveys were not successful in detecting the other two, *cox2* i6 (SPAM11) or *rns* i3 (SPAM12), in any of the 89 isolates of *C. lukuohia*.

Using primers designed for each of the 10 SPAMs present in both species, we identified all 10 SPAMs in each of the 17 tested isolates of *C. huliohia* from Hawai‘i Island. In contrast, PCR showed that each SPAM was heterogeneously and independently distributed among the 89 surveyed isolates of *C. lukuohia*, with no apparent geographic trend (Figure 4).

### Features of the 10 SPAMs in *Ceratocystis huliohia* and *Ceratocystis lukuohia*

**FIGURE 5:**
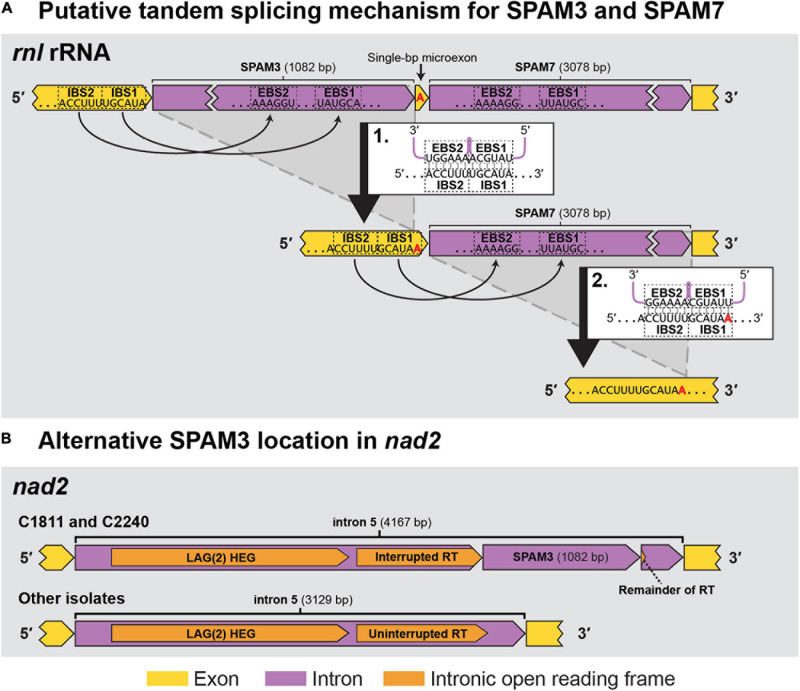
The two observed locations (*rnl* rRNA and *nad2*) for SPAM3 within *Ceratocystis* mitochondrial genomes. **(A)** Proposed intron splicing mechanism for SPAM3 (*rnl* i1) and SPAM7 (*rnl* i2) in *C. huliohia* and *C. lukuohia*, in which SPAM3 interrupts the IBS1 sequence required for splicing of SPAM7; splicing of SPAM3 (step 1) results in the generation of IBS1 for SPAM7, which permits its subsequent removal (step 2). The single-bp exon is indicated in red. EBS = exon binding site; IBS = intron binding site. **(B)** Schematic of *nad2* i5 in *C. fimbriata* isolate C1811 and *C. polychroma* isolate C2240, in which *nad2* i5 is a complex group IIA intron that appears to encode within its domain IV a double-motif LAGLIDADG homing endonuclease [LAG(2)] and a reverse transcriptase (RT) interrupted by an identical copy of SPAM3.

Sequence analyses indicated that seven of the SPAMs were Group II introns, one was a Group I intron, and two were likely autonomous HEGs (Table 3). Most of the 10 SPAMs had typical 5′ and/or 3′ termini (5′ “GUGYG”/3′ “AY” for group II introns and 3′ “G” for group I), but SPAM6 had an unusual “GGGCG” 5′ terminus previously reported in plant (Malek and Knoop, 1998) and yeast (e.g., GenBank AF275271.2, Bullerwell et al., 2003; FN356025.1, Jung et al., 2009), and SPAM3 had a rare 5′ “UUGCG” (Michel et al., 1989; Titov et al., 2019) and a novel 3′ “GC” which has not been previously reported in group II introns. Each SPAM, except SPAM3, encoded a homing endonuclease and/or a reverse transcriptase/maturase complex that could facilitate horizontal transfer (Table 3). SPAM3 appears to instead derive its mobility from a novel ratchet-like splicing mechanism with the neighboring SPAM7 (Figure 5A), somewhat reminiscent of ratchet splicing in spliceosomal introns (Hafez and Hausner, 2015) but unique in that the introns are separated by a single-bp micro exon. SPAM3 is also the only SPAM for which an 100% identical copy was found in a different location than its usual position in *C. lukuohia* and *C. huliohia* in *Ceratocystis polychroma* isolate C2240 (AAC) and *Ceratocystis fimbriata* isolate C1811 (LAC), an identical copy of SPAM3 interrupts *nad2* i5 (rather than *rnl*) and appears to be part of a nested arrangement where it again may not be able to splice independently (Figure 5B). The eight intron SPAMs were easily identified with RNAweasel and are summarized in Table 3. The two SPAMs that we treat as autonomous HEGs were more complicated and are detailed below.

**TABLE 3:** Location and characteristics of 10 mobile mitochondrial elements (SPAMs) found in *Ceratocystis lukuohia* and *Ceratocystis huliohia*.

**SPAM #**	**Location**	**Type**	**Intron encoded proteins (IEPs)**	**Size (bp)**	**5**′ **terminus**	**3**′ **terminus**
SPAM1	*nad2* i1	Group IIA	RT, maturase	2621	Typical; “GTGCG”	Typical; “AC”
SPAM2	*nad6* i2	Group II	RT, maturase	2587	Typical; “GTGCG”	Typical; “AC”
SPAM3	*rnl* i1	Group IIB	None; ORF-less	1082	Unusual; “UUGCG”	Unusual; “GC”
SPAM4	*cox1* i1	Group II	RT, maturase	2757	Typical; “GTGCG”	Typical; “AT”
SPAM5	*cob* i1	Group II	RT, maturase	2492	Typical; “GTGTG”	Typical; “AC”
SPAM6	*nad2* i2	Group IIA	RT, maturase	2740	Unusual; “GGGCG”	Typical; “AT”
SPAM7	*rnl* i2	Group IIB	RT, maturase, HNH HE domain	3078	Typical; “GTGCG”	Typical; “AT”
SPAM8	Inserted within *cox2* i8	Autonomous HEG	Double-motif LAGLIDADG HEG	1159	N/A	N/A
SPAM9	Adjacent to *cob* i5	Autonomous HEG	Double-motif LAGLIDADG HEG	1311	N/A	N/A
SPAM10	*nad4* i2	Group IB	Double-motif LAGLIDADG HEG	1197	N/A	Typical; “G”

The sequence of SPAM8 indicates that it is not an intron but rather a double-motif LAGLIDADG HEG (Dujon, 1989; Stoddard, 2005) that has apparently invaded and interrupts the 5′ end of a single-motif LAGLIDADG HEG in *cox2* i8. Considering that *cox2* i8 orthologs, but not SPAM8, are found in all isolates of *C. lukuohia* and *C. huliohia* (Supplementary Table 1), SPAM8 appears to be not a permanent part of a composite intron but rather an autonomous HEG that moves independently of a ribozyme partner and may continue to invade new sites. This behavior is similar to the putative early ancestors of most intron-embedded HEGs, which invade, co-evolve with, and eventually become integral to their associated introns (Bonocora and Shub, 2009; Edgell, 2009; Megarioti and Kouvelis, 2020). The rest of the *cox2* i8 sequence differs between *C. lukuohia* and *C. huliohia*, indicating that the identical SPAM8 was transferred independently of *cox2* i8.

**FIGURE 6:**
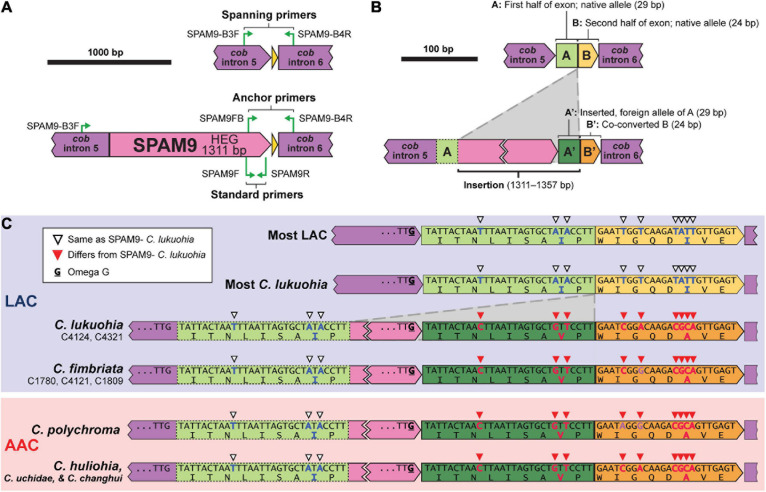
Location, insertion, and co-conversion of SPAM9. **(A)** Proposed arrangements of SPAM9-negative (top) and SPAM9-positive (bottom) alleles of *cob* in *C. lukuohia*. **(B)** Proposed insertion scheme for SPAM9. The *cob* exon is split by the insertion into two regions: “A,” 29 bp, and “B,” 24 bp. SPAM9 is an autonomous homing endonuclease with a slightly different copy of A (A′) on its 3′ end; it inserts in the middle of the exon, splitting A and B, and in doing so co-converts B to B′. **(C)** Observed arrangements of SPAM9 and SPAM9 orthologs in sequenced genomes, with color coding as in panel **(B)**. Full nucleotide sequences and amino acid translations are shown for exon components A and B (green and yellow). Nucleotides and amino acids that match the sequence of *C. huliohia* but not SPAM9-negative *C. lukuohia* are marked by filled red triangles and written with red bolded letters, in contrast to the open triangles and blue bolded letters that match SPAM9-negative *C. lukuohia*. Purple bolded bases in other SPAM9 orthologs are bases that match neither *C. lukuohia* nor *C. huliohia*. The presumed omega-G of *cob* intron 5 is bolded and underlined for each isolate.

SPAM9 also includes a double-motif LAGLIDADG HEG, and neither RNAweasel nor visual inspection conclusively identified it as a group I or group II intron. Additional attempts to characterize it using Infernal 1.1.4 (Nawrocki and Eddy, 2013) and StructRNAfinder (Arias-Carrasco et al., 2018) also failed to identify it as an intron. We chose to also interpret SPAM9 as an autonomous HEG. Due to SPAM9 being flanked by partial copies of the neighboring exon, alignment (and therefore the insertion point) of SPAM9 was somewhat ambiguous. We hypothesize that it inserts into the middle of a 53 bp exon of *cob* (Figure 6B). This leaves the 5′ half of the exon (“A,” 29 bp), which is identical and conserved in all sequenced isolates, upstream of the insertion (Figure 6B). The mobile element appears to include a nearly identical copy of A on its 3′ end, A′, which differs from A by 3 bp (Figures 6B,C). The insertion of the mobile element appears to cause the second half of the exon, B, to change by 6 bp into B′ (Figures 6B,C), a process known as co-conversion that commonly accompanies HEG movement (Belfort et al., 2002; Sanchez-Puerta et al., 2011; Hausner, 2012; Repar and Warnecke, 2017). If this is the true insertion scheme, the presence of a G directly preceding the foreign A′ in the HEG (Figure 6C, bolded and underlined) suggests that the inserted HEG could become part of the upstream intron (*cob* i5) with this G serving as the new omega G, as interpreted in Figures 1, 6. This would cause the foreign A′ and the co-converted B′ to form the newly expressed exon, whereas the native A would become part of *cob* i5. Similar displacements of the native allele with nonidentical copies is seen in other independent HEG insertions (Sethuraman et al., 2009). Importantly, this changes two amino acid positions in the *cob* translation of SPAM9-infected LAC isolates to match the DNA and amino acid sequences of the AAC isolates that SPAM9 presumably came from (Figure 6C), resulting in the aberrant phylogenetic placement of these isolates (Figure 3C). Identical homologs of SPAM9 were found in *C. uchidae* and *C. changhui* (Figure 2B). Non-identical homologs of SPAM9 were found in three isolates of *C. fimbriata* and in *C. polychroma*, each of which had unique sequences with polymorphisms not found in *C. lukuohia* or *C. huliohia* (Figure 6C, bold nucleotides in purple).

### Confirmation of SPAM9 Co-conversion

The association of SPAM9 with the *cob* exon sequence of *C. huliohia* was surveyed in 89 *C. lukuohia* and 17 *C. huliohia* isolates from across Hawai‘i Island using special anchor and spanning PCR primers designed for SPAM9 (Figure 6A). The anchor primers (SPAM9-FB, 5′–CATGCCTTCGGTGACTGGTA–3′; and SPAM9-B4R, 5′–TCCATCACCATCTATTAACCCTACT–3′) produce product only when SPAM9 is present, and the spanning primers (SPAM9-B3F, 5′–TCCCTCCGGGACTCAAATTA–3′ and SPAM9-B4R) produce product only when SPAM9 is absent. All *C. lukuohia* and *C. huliohia* isolates gave results as expected; isolates that gave positive results for SPAM9 in the survey (Figure 4) produced strong products with the anchor primers and absent or very weak products with the spanning primers, and isolates that were negative for SPAM9 produced strong products with the spanning primers and absent or very weak products with the anchor primers. Selected PCR products of each primer pair were sequenced at the ISU DNA Facility using primer SPAM9-B4R, which confirmed exon co-conversion.

## Discussion

Ten mitochondrial regions (SPAMs) found in some but not all isolates of *C. lukuohia* were found in all isolates of *C. huliohia* with 100% identity, apparently as a result of horizontal transfer. The two pathogens are widespread on Hawai‘i Island, and both are wound colonizers that co-occur in diseased sapwood. Frequent horizontal transfer from *C. huliohia* to *C. lukuohia* is a likely explanation for the haphazard distribution of SPAMs in the *C. lukuohia* population. Ancestral transfer of mobile introns has been hypothesized in fungi, but to the authors’ knowledge this is the first report of ongoing transfer of mobile mitochondrial introns between fungal species. The transfer would likely require cytoplasmic connections via hyphal anastomosis, which implies other genetic mitochondrial and nuclear elements (including pathogenicity factors) could be transferred between the species.

### Unique Features of Mitochondrial Genomes and Introns

Introns have been shown to be major contributors to the expansion of fungal mitochondrial genomes in previous studies (Joardar et al., 2012; Férandon et al., 2013; Losada et al., 2014; Mardanov et al., 2014; Kanzi et al., 2016; Wang et al., 2018; Zubaer et al., 2021), and the *Ceratocystis* mitogenomes were large and heavily laden with introns. Our mitochondrial genome sizes (e.g., 161 kb in *C. polychroma*) are relatively large, though not as large as that of fellow family member *E. resinifera* (220 kb) (Zubaer et al., 2018) nor *Morchella crassipes*, which has the largest known mitochondrial genome (531 kb) of an ascomycete (Liu et al., 2020).

An unusual single-bp exon was found between SPAM3 and SPAM7. Small (as few as 3 bp) exons between introns have been observed in fungi (Xavier et al., 2012), and single-bp micro exons have been noted in plants (Guo and Liu, 2015) and metazoans (Osigus et al., 2017), but to the authors’ knowledge this is the first single-bp exon reported in fungi.

SPAM9 and its homologs co-convert the neighboring cob exon in LAC species, and the converted translation products match those of AAC alleles. Co-conversion of insertion sites by independent HEGs may be a survival mechanism to protect against self-cleavage and/or ensure continued mobility (Sethuraman et al., 2009; Zeng et al., 2009). Stable co-conversion requires pervasive and ongoing or recent introgression into a population from outside that population (Repar and Warnecke, 2017), which is consistent with our hypothesis of transfer from *C. huliohia* to *C. lukuohia*. Similar co-conversion of mitochondrial exons in fungi were thought to be mediated by both introns (Zhang et al., 2015; Wang et al., 2018) and HEGs (Sethuraman et al., 2009), and such conversions warrant further study.

### Factors That Support Horizontal Transfer

Each of the 10 SPAMs encoded or worked in tandem with IEPs that are thought to facilitate mobility. Homing endonucleases are common components of mitochondrial introns (Wu and Hao, 2014) and facilitate homing and invasion of intron-minus alleles (Seif et al., 2005; Lang et al., 2007; Freel et al., 2015; Zubaer et al., 2018). The SPAMs each encoded their own HEs or reverse transcriptase/maturases, except for SPAM3 which appears to splice in a unique tandem mechanism with a neighboring intron’s reverse transcriptase/maturase.

It is not clear why only the SPAMs were transferred to *C. lukuohia* and other mitochondrial introns were not. The majority (55/64, 85%) of *C. huliohia* introns were group I introns, many encoding HEGs, but only one of these group I introns (SPAM10) was found in *C. lukuohia*. Evidence suggests that group I introns have been transferred between chloroplasts and mitochondria (Lonergan and Gray, 1994; Turmel et al., 1995), but group I introns are not thought to be long-lived as they may undergo fragmentation to prevent them from reverse splicing (Cech, 1990; Cech et al., 1994).

Group II introns are much rarer than group I introns in fungal mitochondria (Hausner, 2012), but 14% (9/64) of *C. huliohia* introns were group II and seven of them were found in different combinations among isolates of *C. lukuohia.* These seven were the only group II introns observed in *C. lukuohia*. Group II introns, when combined with their encoded reverse transcriptase, are potentially stable as ribonucleoprotein intermediates (Zimmerly et al., 1995a, b; Yang et al., 1996; Guo et al., 1997) which would help facilitate horizontal transfer. Successful horizontal transfer in the two ‘ōhi‘a pathogens appears to be more likely with group II introns that target the 5′ and 3′ ends of protein-coding genes; SPAM1/6, SPAM3/7, SPAM5, and SPAM4 are the first introns in their respective genes, whereas SPAM2 is the last intron of its host gene. In contrast, the only two group II introns of *C. huliohia* not found in *C. lukuohia* were either in the middle of a gene (SPAM11, *cox2* intron 6 of 11) or in a non-protein-coding gene (SPAM12, *rns* i3). Of the non-group II SPAMs, SPAM10 (group I) was the final (3′) intron of its gene, whereas SPAM8 and SPAM9 (both autonomous HEGs) were situated in the middle of genes.

Phylogenetic analyses have suggested that mitochondrial group I introns were widely transferred among the Ascomycota (Lang, 1984; Goddard and Burt, 1999; Mardanov et al., 2014) and that nuclear group I introns have been transferred between unrelated species in Basidiomycota (Hibbett, 1996), in Ascomycota (Holst-Jensen et al., 1999), and between both of these fungal phyla (Gonzalez et al., 1997, 1998, 1999; Rosewich and Kistler, 2000; Mouhamadou et al., 2006). The less-studied Group II introns probably originated as retroelements in bacteria that later moved through fungi to algae and higher plants (Zimmerly et al., 2001) and may have been horizontally transferred in ascomycetous yeasts (Hardy and Clark-Walker, 1991; Rosewich and Kistler, 2000). There are also reports of group I and II introns that were horizontally transferred from fungal and algal sources into broad lineages of eukaryotes including early branching metazoans (Nishimura et al., 2012, 2019; Huchon et al., 2015; Schuster et al., 2017; Dubin et al., 2019). In contrast to these putative ancient events, the horizontal transfer of mitochondrial introns between the two *Ceratocystis* species appears to be recent and ongoing.

Mobile introns likely move between different species through either (1) mitochondrial recombination or (2) transfer via a RNA intermediate (Hardy and Clark-Walker, 1991). We saw no evidence for mtDNA recombination between the species, apart from a single 24 bp region in an intergenic space of a single *C. lukuohia* isolate (C4124) that was otherwise found only in AAC isolates including *C. huliohia*. Hybrid mitochondria would be hindered by protein incompatibility with nuclear-encoded proteins, and many nuclear-encoded proteins were found in mitochondria of the related *C. cacaofunesta* (Ambrosio et al., 2013). Transfer of group II introns via RNA intermediates is more likely since they have stable cytoplasmic intermediaries (ribonucleoprotein particles) and highly efficient reverse-splicing into intron-minus target sites within shared cytoplasm (Lambowitz and Zimmerly, 2011; McNeil et al., 2016). Since group II intron splicing often involves nuclear-encoded genes (McNeil et al., 2016), such transfer may be limited to related fungal species, such as *C. lukuohia* and *C. huliohia*, or certain introns. Independent HEGs, such as SPAM8 and SPAM9, can also be transferred in shared cytoplasm (Koufopanou et al., 2002; Hausner et al., 2014). The presence of a co-conversion tract associated with SPAM9 may have been transferred by direct contact and crossing-over of mitochondrial genomes (Sanchez-Puerta et al., 2011; Repar and Warnecke, 2017), but such crossing over would seem to be more difficult and rare than the cytoplasmic transfer of stable intermediates such as an HEG.

### Possible Impact of Mitochondrial Introns

Mitochondrial introns can negatively affect expression of the genes they insert into by reducing transcription efficiency (Werren, 2011; Belfort, 2017; Rudan et al., 2018; Castell-Miller and Samac, 2019). Mitochondrial introns in fungal pathogens can cause hypovirulence (Baidyaroy et al., 2011) and may be present in higher numbers in less-aggressive isolates (Bates et al., 1993; Sethuraman et al., 2008). Hosts can eventually evolve to accommodate the deleterious effects of long-established introns by co-opting some of these elements as gene regulatory elements (Rudan et al., 2018). However, the multiple introns recently incorporated into the mitochondrial genomes of *C. lukuohia*, many in respiration-related genes, may have introduced splicing as a rate limiting step and thus introduced respiratory inefficiencies. It is interesting that *C. huliohia* and other AAC species are much more intron-laden and less aggressive pathogens than species in the LAC. Besides *C. lukuohia*, the largest (and most intron-laden) mitochondrial genomes in the LAC were in the *C. fimbriata* strains that attack root crops (ex *Xanthosoma*, *Syngonium*, and *Ipomoea*) and are routinely mixed and spread on cutting tools during vegetative propagation, but the fewest introns were in the most aggressive South American strains (ex *Eucalyptus* and *Mangifera*) (Thorpe et al., 2005; Oliveira et al., 2016; Li et al., 2017).

### Geographic Heterogeneity and Recent Horizontal Transfer of *Ceratocystis lukuohia* Mitochondrial Elements

The PCR surveys with SPAM-specific primers showed that each SPAM element was haphazardly distributed among the 89 isolates of *C. lukuohia* but present in each of the 17 isolates of *C. huliohia*. It is assumed that *C. lukuohia* is a recent (within the last few decades) introduction to Hawai‘i, and the fungus shows very limited nuclear (Barnes et al., 2018) or mitochondrial diversity, besides the SPAMs. *C. huliohia* may have gone undetected for longer, but it too shows very limited genetic variation (Barnes et al., 2018; Heller et al., 2019), and its mitochondrial genomes were essentially identical. *C. lukuohia* is not known outside of Hawai‘i, but its close relative *C. fimbriata* ex *Syngonium* is a widely distributed genotype that has been in Hawai‘i greenhouses for more than three decades (Thorpe et al., 2005). The *Syngonium* genotype had homologs of several SPAMs, but they differ in sequence identity (80–98%) and are therefore not the source of the SPAMs in *C. lukuohia*.

Isolates of *C. lukuohia* in areas of Hawai‘i Island where both *C. lukuohia* and *C. huliohia* occur had more SPAMs than isolates from areas where only *C. lukuohia* is known. Isolates with the greatest number of SPAMs were from trees that had been recently wounded by earlier sampling or road construction. Such wounds would promote interaction between the two pathogens. In contrast, *C. huliohia* has not been found in the Kohala area, the northernmost and most aggressive outbreak area on Hawai‘i Island, and *C. lukuohia* isolates from Kohala had the fewest SPAMs. This suggests SPAMs may progressively accumulate as populations of *C. lukuohia* interact with those of *C. huliohia* over time.

The sequences of the SPAM elements in *C. lukuohia* are identical to *C. huliohia* elements and similar to other AAC elements, strongly suggesting that the SPAMs were horizontally transferred from *C. huliohia.* Movement of the SPAMs during a sexual cross is unlikely because *C. lukuohia* and *C. huliohia* are sexually incompatible and mitochondrial transmission is uniparental (female inherited) in *Ceratocystis* (Harrington, unpublished data). The haphazard geographic distribution of SPAMs in *C. lukuohia* suggests the transfer was not a single early event. Sporadic and transient hyphal fusion events (anastomosis) with *C. huliohia* on wounds or in sapwood of trees killed by *C. lukuohia* would allow cytoplasmic exchange (Hausner, 2012), including exchange of whole mitochondria and intron RNA intermediaries. In the Mucoromycota fungus *Rhizophagus irregularis*, the anastomosed hyphae of genetically diverse individuals produced spores with mixes of both parental mitochondria, but only homoplasmic spores germinated (de la Providencia et al., 2013). In the ascomycetes *Neurospora crassa* (Charter et al., 1993) and *Ophiostoma novo-ulmi* (Hausner et al., 2006), hyphal anastomoses allowed infective plasmid-like elements to transfer through the cytoplasm to infect the mitochondria of other strains. Heteroplasmic cytoplasm with mitochondria of both *C. lukuohia* and *C. huliohia* could allow for exchange of mobile introns and HEGs, even if *C. huliohia* mitochondria were incompatible with the nuclear-encoded genes of *C. lukuohia* (Ambrosio et al., 2013) and did not ultimately persist in progeny.

It is also possible that horizontal transfer of the 10 SPAMs to *C. lukuohia* occurred once or occurs rarely, and that the haphazard nature of the distribution of the SPAMs in *C. lukuohia* is mostly the result of intraspecific transfers to other *C. lukuohia* strains through sexual crossing or anastomosis. Selection for rapid division of SPAM-free mitochondria at hyphal tips or the relative infectivity of some of the SPAMs could affect the heterogeneity of the SPAMs in individual thalli and populations.

Intraspecific intron diversity is not unprecedented in the family Ceratocystidaceae. *E. resinifera* mitogenomes from Canada and Europe differed in the presence of four mtDNA introns (Zubaer et al., 2018). However, our *C. lukuohia* isolates comprise a single population on an island that presumably originated from a single recent introduction, yet the *C. lukuohia* populations have more than twice as many intron polymorphisms as observed in *E. resinifera*. The transfer of SPAMs is in stark contrast to “recent” and “frequent” horizontal transfer measured in many millions of years in yeasts (Goddard and Burt, 1999). The SPAM elements are all 100% identical in *C. lukuohia* and *C. huliohia*, again in contrast with “recent” horizontal transfer inferred from 96% identity in yeasts (Hardy and Clark-Walker, 1991).

We propose that the 10 SPAM elements were horizontally transferred very recently and may continue to be transferred where *C. lukuohia* co-occurs with *C. huliohia*. Culture studies should be undertaken to determine whether *C. lukuohia* and *C. huliohia* are able to undergo hyphal fusion and to experimentally demonstrate the horizontal transfer of SPAM elements. Also, the fitness and pathogenicity of *C. lukuohia* isolates with varying intron loads should be measured to determine whether intron load attenuates pathogenicity or other fitness characters. Other selfish genetic elements can also be horizontally transferred in fungi, including mycoviruses, mitochondrial plasmids, and transposons (Rosewich and Kistler, 2000; Fitzpatrick, 2012; Hausner, 2012; Sandor et al., 2018), and the possibility of exchange of such elements or other genetic factors between the two pathogens warrants further study.

## Data Availability Statement

The datasets presented in this study can be found in online repositories. The names of the repository/repositories and accession number(s) can be found in the article/Supplementary Material.

## Author Contributions

All authors contributed to the work: CM worked under the supervision of TH, and AW under the supervision of GH. TH obtained the fungal isolates. CM conducted the majority of dataset assembly and analysis. CM and TH contributed to the design of the project. CM assembled the final version of the manuscript. All authors contributed toward the analysis and interpretation of data and worked on the manuscript.

## Conflict of Interest

The authors declare that the research was conducted in the absence of any commercial or financial relationships that could be construed as a potential conflict of interest.

## Footnotes

1 http://megasun.bch.umontreal.ca/RNAweasel/

2 http://purl.org/phylo/treebase/phylows/study/TB2:S27637

3 http://purl.org/phylo/treebase/phylows/study/TB2:S27638

4 http://purl.org/phylo/treebase/phylows/study/TB2:S27636

5 https://urs.earthdata.nasa.gov

## References

[B1] Aguileta G. De Vienne D. M. Ross O. N. Hood M. E. Giraud T. Petit E. (2014). High variability of mitochondrial gene order among fungi. *Genome Biol. Evol.* 6 451–465. 10.1093/gbe/evu028 24504088 PMC3942027

[B2] Ambrosio A. B. do Nascimento L. C. Oliveira B. V. Teixeira P. J. Tiburcio R. A. Thomazella D. P. (2013). Global analyses of *Ceratocystis cacaofunesta* mitochondria: from genome to proteome. *BMC Genomics* 14:91. 10.1186/1471-2164-14-91 23394930 PMC3605234

[B3] Arias-Carrasco R. Vásquez-Morán Y. Nakaya H. I. Maracaja-Coutinho V. (2018). StructRNAfinder: an automated pipeline and web server for RNA families prediction. *BMC Bioinformatics* 19:55. 10.1186/s12859-018-2052-2 29454313 PMC5816368

[B4] Baidyaroy D. Hausner G. Hafez M. Michel F. Fulbright D. W. Bertrand H. (2011). A 971-bp insertion in the rns gene is associated with mitochondrial hypovirulence in a strain of Cryphonectria parasitica isolated from nature. *Fungal Genet. Biol.* 48 775–783. 10.1016/j.fgb.2011.05.006 21601643

[B5] Baker C. J. Harrington T. C. Krauss U. Alfenas A. C. (2003). Genetic variability and host specialization in the Latin American clade of *Ceratocystis fimbriata*. *Phytopathology* 93 1274–1284. 10.1094/phyto.2003.93.10.1274 18944327

[B6] Barnes I. Fourie A. Wingfield M. J. Harrington T. C. McNew D. L. Sugiyama L. S. (2018). New Ceratocystis species associated with rapid death of *Metrosideros polymorpha* in Hawai’i. *Persoonia* 40 154–181. 10.3767/persoonia.2018.40.07 30505000 PMC6146641

[B7] Bates M. R. Buck K. W. Brasier C. M. (1993). Molecular relationships of the mitochondrial DNA of *Ophiostoma ulmi* and the NAN and EAN races of O. novo-ulmi determined by restriction fragment length polymorphisms. *Mycol. Res.* 97 1093–1100. 10.1016/s0953-7562(09)80510-8

[B8] Belfort M. (2017). Mobile self-splicing introns and inteins as environmental sensors. *Curr. Opin. Microbiol.* 38 51–58. 10.1016/j.mib.2017.04.003 28482231 PMC5671916

[B9] Belfort M. Derbyshire V. Parker M. M. Cousineau B. Lambowitz A. M. (2002). “Mobile introns: pathways and proteins,” in *Mobile DNA II*, eds Craig N. L. Craigie R. Gellert M. Lambowitz A. M. (Washington, DC: ASM Press), 761–783. 10.1128/9781555817954.ch31

[B10] Belfort M. Perlman P. S. (1995). Mechanisms of intron mobility. *J. Biol. Chem.* 270 30237–30240. 10.1074/jbc.270.51.30237 8530436

[B11] Bonocora R. P. Shub D. A. (2009). A likely pathway for formation of mobile group I introns. *Curr. Biol.* 19 223–228. 10.1016/j.cub.2009.01.033 19200727 PMC2856452

[B12] Bullerwell C. E. Leigh J. Forget L. Lang B. F. (2003). A comparison of three fission yeast mitochondrial genomes. *Nucleic Acids Res.* 31 759–768. 10.1093/nar/gkg134 12527786 PMC140500

[B13] Castell-Miller C. V. Samac D. A. (2019). Sensitivity of *Bipolaris oryzae* isolates pathogenic on cultivated wild rice to the quinone outside inhibitor azoxystrobin. *Plant Dis.* 103 1910–1917. 10.1094/pdis-12-18-2267-re 31140926

[B14] Cech T. R. (1990). Self-splicing of group I introns. *Annu. Rev. Biochem.* 59 543–568. 10.1146/annurev.bi.59.070190.002551 2197983

[B15] Cech T. R. Damberger S. H. Gutell R. R. (1994). Representation of the secondary and tertiary structure of group I introns. *Nat. Struct. Biol.* 1 273–280. 10.1038/nsb0594-273 7545072

[B16] Charter N. W. Buck K. W. Brasier C. M. (1993). De-novo generation of mitochondrial DNA plasmids following cytoplasmic transmission of a degenerative disease in Ophiostoma novo-ulmi. *Curr. Genet.* 24 505–514. 10.1007/bf00351714 8299172

[B17] Cheeseman K. Ropars J. Renault P. Dupont J. Gouzy J. Branca A. (2014). Multiple recent horizontal transfers of a large genomic region in cheese making fungi. *Nat. Commun.* 5:2876. 10.1038/ncomms3876 PMC3896755 24407037

[B18] Chevalier B. S. Stoddard B. L. (2001). Homing endonucleases: structural and functional insight into the catalysts of intron/intein mobility. *Nucleic Acids Res.* 29 3757–3774. 10.1093/nar/29.18.3757 11557808 PMC55915

[B19] de la Providencia I. E. Nadimi M. Beaudet D. Rodriguez Morales G. Hijri M. (2013). Detection of a transient mitochondrial DNA heteroplasmy in the progeny of crossed genetically divergent isolates of Arbuscular mycorrhizal fungi. *New Phytol.* 200 211–221. 10.1111/nph.12372 23790215

[B20] Dierckxsens N. Mardulyn P. Smits G. (2017). NOVOPlasty: de novo assembly of organelle genomes from whole genome data. *Nucleic Acids Res.* 45:e18. 10.1093/nar/gkw955 PMC5389512 28204566

[B21] Dubin A. Chi S. I. Emblem Å Moum T. Johansen S. D. (2019). Deep-water sea anemone with a two-chromosome mitochondrial genome. *Gene* 692 195–200. 10.1016/j.gene.2018.12.074 30641219

[B22] Dujon B. (1989). Group I introns as mobile genetic elements: facts and mechanistic speculations—a review. *Gene* 82 91–114. 10.1016/0378-1119(89)90034-6 2555264

[B23] Edgell D. R. (2009). Selfish DNA: homing endonucleases find a home. *Curr. Biol.* 19 R115–R117. 19211047 10.1016/j.cub.2008.12.019

[B24] Engelbrecht C. J. Harrington T. C. (2005). Intersterility, morphology and taxonomy of *Ceratocystis fimbriata* on sweet potato, cacao and sycamore. *Mycologia* 97 57–69. 10.3852/mycologia.97.1.57 16389957

[B25] Férandon C. Xu J. Barroso G. (2013). The 135 kbp mitochondrial genome of *Agaricus bisporus* is the largest known eukaryotic reservoir of group I introns and plasmid-related sequences. *Fungal Genet. Biol.* 55 85–91. 10.1016/j.fgb.2013.01.009 23428625

[B26] Feurtey A. Stukenbrock E. H. (2018). Interspecific gene exchange as a driver of adaptive evolution in fungi. *Annu. Rev. Microbiol.* 72 377–398. 10.1146/annurev-micro-090817-062753 29927707

[B27] Fitzpatrick D. A. (2012). Horizontal gene transfer in fungi. *FEMS Microbiol. Lett.* 329 1–8. 10.1111/j.1574-6968.2011.02465.x 22112233

[B28] Freel K. C. Friedrich A. Schacherer J. (2015). Mitochondrial genome evolution in yeasts: an all-encompassing view. *FEMS Yeast Res.* 15:fov023. 10.1093/femsyr/fov023 25969454

[B29] Goddard M. R. Burt A. (1999). Recurrent invasion and extinction of a selfish gene. *Proc. Natl. Acad. Sci. U.S.A.* 96 13880–13885. 10.1073/pnas.96.24.13880 10570167 PMC24159

[B30] Gonzalez P. Barroso G. Labarère J. (1997). DNA sequence and secondary structure of the mitochondrial small subunit ribosomal RNA coding region including a group-IC2 intron from the cultivated basidiomycete *Agrocybe aegerita*. *Gene* 184 55–63. 10.1016/s0378-1119(96)00573-2 9016953

[B31] Gonzalez P. Barroso G. Labarère J. (1998). Molecular analysis of the split cox1 gene from the Basidiomycota *Agrocybe aegerita*: relationship of its introns with homologous Ascomycota introns and divergence levels from common ancestral copies. *Gene* 220 45–53. 10.1016/s0378-1119(98)00421-1 9767103

[B32] Gonzalez P. Barroso G. Labarère J. (1999). Molecular gene organisation and secondary structure of the mitochondrial large subunit ribosomal RNA from the cultivated Basidiomycota *Agrocybe aegerita*: a 13 kb gene possessing six unusual nucleotide extensions and eight introns. *Nucleic Acids Res.* 27 1754–1761. 10.1093/nar/27.7.1754 10076008 PMC148380

[B33] Gray M. W. (1998). Mass migration of a group I intron: promiscuity on a grand scale. *Proc. Natl. Acad. Sci. U.S.A.* 95 14003–14005. 10.1073/pnas.95.24.14003 9826641 PMC33921

[B34] Guindon S. Dufayard J. F. Lefort V. Anisimova M. Hordijk W. Gascuel O. (2010). New algorithms and methods to estimate maximum-likelihood phylogenies: assessing the performance of PhyML 3.0. *Syst. Biol.* 59 307–321. 10.1093/sysbio/syq010 20525638

[B35] Guo H. Zimmerly S. Perlman P. S. Lambowitz A. M. (1997). Group II intron endonucleases use both RNA and protein subunits for recognition of specific sequences in double-stranded DNA. *EMBO J.* 16 6835–6848. 10.1093/emboj/16.22.6835 9362497 PMC1170287

[B36] Guo L. Liu C. M. (2015). A single-nucleotide exon found in *Arabidopsis*. *Sci. Rep.* 5:18087. 10.1038/srep18087 PMC4674806 26657562

[B37] Hafez M. Hausner G. (2012). Homing endonucleases: DNA scissors on a mission. *Genome* 55 553–569. 10.1139/g2012-049 22891613

[B38] Hafez M. Hausner G. (2015). Convergent evolution of twintron-like configurations: one is never enough. *RNA Biol.* 12 1275–1288. 10.1080/15476286.2015.1103427 26513606 PMC4829276

[B39] Hardy C. M. Clark-Walker G. D. (1991). Nucleotide sequence of the COX1 gene in *Kluyveromyces lactis* mitochondrial DNA: evidence for recent horizontal transfer of a group II intron. *Curr. Genet.* 20 99–114. 10.1007/bf00312772 1657415

[B40] Hausner G. (2012). “Introns, mobile elements, and plasmids,” in *Organelle Genetics*, ed. Bullerwell C. E. (Heidelberg: Springer-Verlag), 329–357. 10.1007/978-3-642-22380-8_13

[B41] Hausner G. Hafez M. Edgell D. R. (2014). Bacterial group I introns: mobile RNA catalysts. *Mobile DNA* 5:8. 10.1186/1759-8753-5-8 24612670 PMC3984707

[B42] Hausner G. Nummy K. A. Bertrand H. (2006). Asexual transmission, non-suppressiveness and meiotic extinction of small plasmid-like derivatives of the mitochondrial DNA in Neurospora crassa. *Fungal Genet. Biol.* 43 90–101. 10.1016/j.fgb.2005.10.004 16386438

[B43] Heller W. P. Hughes M. A. Luiz B. C. Brill E. Friday J. B. Williams A. M. (2019). First report of *Ceratocystis huliohia* causing mortality of *Metrosideros polymorpha* trees on the Island of Kaua‘i, Hawai‘i USA. *Forest Pathol.* 49:e12546. 10.1111/efp.12546

[B44] Hibbett D. S. (1996). Phylogenetic evidence for horizontal transmission of group I introns in the nuclear ribosomal DNA of mushroom-forming fungi. *Mol. Biol. Evol.* 13 903–917. 10.1093/oxfordjournals.molbev.a025658 8751999

[B45] Holst-Jensen A. Vaage M. Schumacher T. Johansen S. (1999). Structural characteristics and possible horizontal transfer of group I introns between closely related plant pathogenic fungi. *Mol. Biol. Evol.* 16 114–126. 10.1093/oxfordjournals.molbev.a026031 10331256

[B46] Huchon D. Szitenberg A. Shefer S. Ilan M. Feldstein T. (2015). Mitochondrial group I and group II introns in the sponge orders *Agelasida* and *Axinellida*. *BMC Evol. Biol.* 15:278. 10.1186/s12862-015-0556-1 26653218 PMC4676843

[B47] Hughes M. A. Juzwik J. Harrington T. Keith L. (2020). Pathogenicity, symptom development and colonization of *Metrosideros polymorpha* by Ceratocystis lukuohia. *Plant Dis.* 104 2233–2241. 10.1094/pdis-09-19-1905-re 32552282

[B48] Joardar V. Abrams N. F. Hostetler J. Paukstelis P. J. Pakala S. Pakala S. B. (2012). Sequencing of mitochondrial genomes of nine *Aspergillus* and *Penicillium* species identifies mobile introns and accessory genes as main sources of genome size variability. *BMC Genomics* 13:698. 10.1186/1471-2164-13-698 23234273 PMC3562157

[B49] Johnson J. A. Harrington T. C. Engelbrecht C. J. (2005). Phylogeny and taxonomy of the North American clade of the *Ceratocystis fimbriata* complex. *Mycologia* 97 1067–1092. 10.3852/mycologia.97.5.1067 16596958

[B50] Jung P. P. Schacherer J. Souciet J. L. Potier S. Wincker P. De Montigny J. (2009). The complete mitochondrial genome of the yeast *Pichia sorbitophila*. *FEMS Yeast Res.* 9 903–910. 10.1111/j.1567-1364.2009.00540.x 19594828

[B51] Kanzi A. M. Wingfield B. D. Steenkamp E. T. Naidoo S. van der Merwe N. A. (2016). Intron derived size polymorphism in the mitochondrial genomes of closely related *Chrysoporthe* species. *PLoS One* 11:e0156104. 10.1371/journal.pone.0156104 27272523 PMC4894602

[B52] Keith L. M. Hughes R. F. Sugiyama L. S. Heller W. P. Bushe B. C. Friday J. B. (2015). First report of *Ceratocystis* wilt on ‘Ōhi‘a (*Metrosideros polymorpha*). *Plant Dis.* 99 1276. 10.1094/pdis-12-14-1293-pdn

[B53] Koufopanou V. Goddard M. R. Burt A. (2002). Adaptation for horizontal transfer in a homing endonuclease. *Mol. Biol. Evol.* 19 239–246. 10.1093/oxfordjournals.molbev.a004077 11861883

[B54] Lambowitz A. M. Perlman P. S. (1990). Involvement of aminoacyl-tRNA synthetases and other proteins in group I and group II intron splicing. *Trends Biochem. Sci.* 15 440–444. 10.1016/0968-0004(90)90283-h 2278103

[B55] Lambowitz A. M. Zimmerly S. (2011). Group II introns: mobile ribozymes that invade DNA. *Cold Spring Harb. Perspect. Biol.* 3:a003616. 10.1101/cshperspect.a003616 20463000 PMC3140690

[B56] Lanfear R. Calcott B. Ho S. Y. Guindon S. (2012). PartitionFinder: combined selection of partitioning schemes and substitution models for phylogenetic analyses. *Mol. Biol. Evol.* 29 1695–1701. 10.1093/molbev/mss020 22319168

[B57] Lanfear R. Frandsen P. B. Wright A. M. Senfeld T. Calcott B. (2017). PartitionFinder 2: new methods for selecting partitioned models of evolution for molecular and morphological phylogenetic analyses. *Mol. Biol. Evol.* 34 772–773. 28013191 10.1093/molbev/msw260

[B58] Lang B. F. (1984). The mitochondrial genome of the fission yeast *Schizosaccharomyces pombe*: highly homologous introns are inserted at the same position of the otherwise less conserved cox1 genes in *Schizosaccharomyces pombe* and *Aspergillus nidulans*. *EMBO J.* 3 2129–2136. 10.1002/j.1460-2075.1984.tb02102.x 6092057 PMC557654

[B59] Lang B. F. Laforest M. J. Burger G. (2007). Mitochondrial introns: a critical view. *Trends Genet.* 23 119–125. 10.1016/j.tig.2007.01.006 17280737

[B60] Li Q. Harrington T. C. McNew D. Li J. (2017). Ceratocystis uchidae, a new species on *Araceae* in Hawaii and Fiji. *Mycoscience* 58 398–412. 10.1016/j.myc.2017.06.001

[B61] Liu F. F. Barnes I. Roux J. Wingfield M. J. Chen S. (2018). Molecular phylogenetics and microsatellite analysis reveal a new pathogenic *Ceratocystis* species in the Asian-Australian clade. *Plant Pathol.* 67 1097–1113. 10.1111/ppa.12820

[B62] Liu W. Cai Y. Zhang Q. Shu F. Chen L. Ma X. (2020). Subchromosome-scale nuclear and complete mitochondrial genome characteristics of *Morchella crassipes*. *Int. J. Mol. Sci.* 21:483. 10.3390/ijms21020483 31940908 PMC7014384

[B63] Lonergan K. M. Gray M. W. (1994). The ribosomal RNA gene region in *Acanthamoeba castellanii* mitochondrial DNA: a case of evolutionary transfer of introns between mitochondria and plastids? *J. Mol. Biol.* 239 476–499. 10.1006/jmbi.1994.1390 8006963

[B64] Losada L. Pakala S. B. Fedorova N. D. Joardar V. Shabalina S. A. Hostetler J. (2014). Mobile elements and mitochondrial genome expansion in the soil fungus and potato pathogen *Rhizoctonia solani* AG-3. *FEMS Microbiol. Lett.* 352 165–173. 10.1111/1574-6968.12387 24461055

[B65] Malek O. Knoop V. (1998). Trans-splicing group II introns in plant mitochondria: the complete set of cis-arranged homologs in ferns, fern allies, and a hornwort. *RNA* 4 1599–1609. 10.1017/s1355838298981262 9848656 PMC1369728

[B66] Mardanov A. V. Beletsky A. V. Kadnikov V. V. Ignatov A. N. Ravin N. V. (2014). The 203 kbp mitochondrial genome of the phytopathogenic fungus *Sclerotinia borealis* reveals multiple invasions of introns and genomic duplications. *PLoS One* 9:e107536. 10.1371/journal.pone.0107536 25216190 PMC4162613

[B67] Matsuura M. Noah J. W. Lambowitz A. M. (2001). Mechanism of maturase-promoted group II intron splicing. *EMBO J.* 20 7259–7270. 10.1093/emboj/20.24.7259 11743002 PMC125332

[B68] McNeil B. A. Semper C. Zimmerly S. (2016). Group II introns: versatile ribozymes and retroelements. *Wiley Interdiscip. Rev. RNA* 7 341–355. 10.1002/wrna.1339 26876278

[B69] Megarioti A. H. Kouvelis V. N. (2020). The coevolution of fungal mitochondrial introns and their Homing Endonucleases (GIY-YIG and LAGLIDADG). *Genome Biol. Evol.* 12 1337–1354. 10.1093/gbe/evaa126 32585032 PMC7487136

[B70] Mehrabi R. Bahkali A. H. Abd-Elsalam K. A. Moslem M. Ben M. Barek S. (2011). Horizontal gene and chromosome transfer in plant pathogenic fungi affecting host range. *FEMS Microbiol. Rev.* 35 542–554. 10.1111/j.1574-6976.2010.00263.x 21223323

[B71] Michel F. Kazuhiko U. Haruo O. (1989). Comparative and functional anatomy of group II catalytic introns–a review. *Gene* 82 5–30. 10.1016/0378-1119(89)90026-7 2684776

[B72] Mortenson L. A. Hughes R. F. Friday J. B. Keith L. M. Barbosa J. M. Friday N. J. (2016). Assessing spatial distribution, stand impacts and rate of *Ceratocystis fimbriata* induced ‘ōhi ‘a (*Metrosideros polymorpha*) mortality in a tropical wet forest, Hawai ‘i Island, USA. *Forest Ecol. Manag.* 377 83–92. 10.1016/j.foreco.2016.06.026

[B73] Mouhamadou B. Férandon C. Barroso G. Labarère J. (2006). The mitochondrial apocytochrome b genes of two *Agrocybe* species suggest lateral transfers of group I homing introns among phylogenetically distant fungi. *Fungal Genet. Biol.* 43 135–145. 10.1016/j.fgb.2005.07.001 16504553

[B74] Mullineux S. T. Costa M. Bassi G. S. Michel F. Hausner G. (2010). A group II intron encodes a functional LAGLIDADG homing endonuclease and self-splices under moderate temperature and ionic conditions. *RNA* 16 1818–1831. 10.1261/rna.2184010 20656798 PMC2924541

[B75] Nawrocki E. P. Eddy S. R. (2013). Infernal 1.1: 100-fold faster RNA homology searches. *Bioinformatics* 29 2933–2935. 10.1093/bioinformatics/btt509 24008419 PMC3810854

[B76] Nishimura Y. Kamikawa R. Hashimoto T. Inagaki Y. (2012). Separate origins of group I introns in two mitochondrial genes of the katablepharid *Leucocryptos marina*. *PLoS One* 7:e37307. 10.1371/journal.pone.0037307 22606358 PMC3350498

[B77] Nishimura Y. Shiratori T. Ishida K. I. Hashimoto T. Ohkuma M. Inagaki Y. (2019). Horizontally-acquired genetic elements in the mitochondrial genome of a centrohelid *Marophrys* sp. SRT127. *Sci. Rep.* 9:4850. 10.1038/s41598-019-41238-6 PMC6425028 30890720

[B78] Oliveira L. S. Damacena M. B. Guimarães L. M. Siqueira D. L. Alfenas A. C. (2016). *Ceratocystis fimbriata* isolates on *Mangifera indica* have different levels of aggressiveness. *Eur. J. Plant Pathol.* 145 847–856. 10.1007/s10658-016-0873-2

[B79] Osigus H. J. Eitel M. Schierwater B. (2017). Deep RNA sequencing reveals the smallest known mitochondrial micro exon in animals: the placozoan cox1 single base pair exon. *PLoS One* 12:e0177959. 10.1371/journal.pone.0177959 28542197 PMC5436844

[B80] Repar J. Warnecke T. (2017). Mobile introns shape the genetic diversity of their host genes. *Genetics* 205 1641–1648. 10.1534/genetics.116.199059 28193728 PMC5378118

[B81] Ronquist F. Teslenko M. Van Der Mark P. Ayres D. L. Darling A. Höhna S. (2012). MrBayes 3.2: efficient Bayesian phylogenetic inference and model choice across a large model space. *Syst. Biol.* 61 539–542. 10.1093/sysbio/sys029 22357727 PMC3329765

[B82] Rosewich U. L. Kistler H. C. (2000). Role of horizontal gene transfer in the evolution of fungi. *Annu. Rev. Phytopathol.* 38 325–363. 10.1146/annurev.phyto.38.1.325 11701846

[B83] Roy K. Ewing C. P. Hughes M. A. Keith L. Bennett G. M. (2019). Presence and viability of *Ceratocystis lukuohia* in ambrosia beetle frass from Rapid ‘Ōhi‘a Death-affected *Metrosideros polymorpha* trees on Hawai‘i Island. *Forest Pathol.* 49:e12476. 10.1111/efp.12476

[B84] Roy K. Jaenecke K. A. Peck R. W. (2020). Ambrosia beetle (Coleoptera: Curculionidae) communities and frass production in ‘Ōhi‘a (Myrtales: Myrtaceae) infected with *Ceratocystis* (Microascales: Ceratocystidaceae) fungi responsible for Rapid ‘Ōhi‘a Death. *Environ. Entomol.* 49 1345–1354. 10.1093/ee/nvaa108 33315073

[B85] Rudan M. Dib P. B. Musa M. Kanunnikau M. Sobočanec S. Rueda D. (2018). Normal mitochondrial function in *Saccharomyces cerevisiae* has become dependent on inefficient splicing. *eLife* 7:e35330. 10.7554/eLife.35330 PMC5898908 29570052

[B86] Sanchez-Puerta M. V. Abbona C. C. Zhuo S. Tepe E. J. Bohs L. Olmstead R. G. (2011). Multiple recent horizontal transfers of the cox1 intron in Solanaceae and extended co-conversion of flanking exons. *BMC Evol. Biol.* 11:277. 10.1186/1471-2148-11-277 21943226 PMC3192709

[B87] Sandor S. Zhang Y. Xu J. (2018). Fungal mitochondrial genomes and genetic polymorphisms. *Appl. Microbiol. Biotechnol.* 102 9433–9448. 10.1007/s00253-018-9350-5 30209549

[B88] Schuster A. Lopez J. V. Becking L. E. Kelly M. Pomponi S. A. Wörheide G. (2017). Evolution of group I introns in Porifera: new evidence for intron mobility and implications for DNA barcoding. *BMC Evol. Biol.* 17:82. 10.1186/s12862-017-0928-9 28320321 PMC5360047

[B89] Seif E. Leigh J. Liu Y. Roewer I. Forget L. Lang B. F. (2005). Comparative mitochondrial genomics in zygomycetes: bacteria-like RNase P RNAs, mobile elements and a close source of the group I intron invasion in angiosperms. *Nucleic Acids Res.* 33 734–744. 15689432 10.1093/nar/gki199 PMC548346

[B90] Sellem C. H. Belcour L. (1997). Intron open reading frames as mobile elements and evolution of a group I intron. *Mol. Biol. Evol.* 14 518–526. 10.1093/oxfordjournals.molbev.a025788 9159929

[B91] Sethuraman J. Majer A. Friedrich N. C. Edgell D. R. Hausner G. (2009). Genes within genes: multiple LAGLIDADG homing endonucleases target the ribosomal protein S3 gene encoded within an rnl group I intron of Ophiostoma and related taxa. *Mol. Biol. Evol.* 26 2299–2315. 10.1093/molbev/msp145 19597163

[B92] Sethuraman J. Okoli C. V. Majer A. Corkery T. L. Hausner G. (2008). The sporadic occurrence of a group I intron-like element in the mtDNA rnl gene of Ophiostoma novo-ulmi subsp. americana. *Mycol. Res.* 112 564–582. 10.1016/j.mycres.2007.11.017 18406119

[B93] Stoddard B. L. (2005). Homing endonuclease structure and function. *Q. Rev. Biophys.* 38 49–95. 10.1017/s0033583505004063 16336743

[B94] Stoddard B. L. (2011). Homing endonucleases: from microbial genetic invaders to reagents for targeted DNA modification. *Structure* 19 7–15. 10.1016/j.str.2010.12.003 21220111 PMC3038549

[B95] Thorpe D. J. Harrington T. C. Uchida J. Y. (2005). Pathogenicity, internal transcribed spacer-rDNA variation, and human dispersal of *Ceratocystis fimbriata* on the family Araceae. *Phytopathology* 95 316–323. 10.1094/phyto-95-0316 18943126

[B96] Titov I. Kobalo N. Vorobyev D. Kulikov A. (2019). A bioinformatic method for identifying Group II introns in organella (sic) genomes. *Front. Genet.* 10:1135. 10.3389/fgene.2019.01135 31798632 PMC6867995

[B97] Toor N. Zimmerly S. (2002). Identification of a family of group II introns encoding LAGLIDADG ORFs typical of group I introns. *RNA* 8 1373–1377. 10.1017/s1355838202023087 12458791 PMC1370344

[B98] Turmel M. Côté V. Otis C. Mercier J. P. Gray M. W. Lonergan K. M. (1995). Evolutionary transfer of ORF-containing group I introns between different subcellular compartments (chloroplast and mitochondrion). *Mol. Biol. Evol.* 12 533–545. 7659010 10.1093/oxfordjournals.molbev.a040234

[B99] Vicens Q. Paukstelis P. J. Westhof E. Lambowitz A. M. Cech T. R. (2008). Toward predicting self-splicing and protein-facilitated splicing of group I introns. *RNA* 14 2013–2029. 10.1261/rna.1027208 18768647 PMC2553746

[B100] Wallweber G. J. Mohr S. Rennard R. Caprara M. G. Lambowitz A. M. (1997). Characterization of Neurospora mitochondrial group I introns reveals different CYT-18 dependent and independent splicing strategies and an alternative 3′ splice site for an intron ORF. *RNA* 3 114–131. 9042940 PMC1369467

[B101] Wang L. Zhang S. Li J. H. Zhang Y. J. (2018). Mitochondrial genome, comparative analysis and evolutionary insights into the entomopathogenic fungus *Hirsutella thompsonii*. *Environ. Microbiol.* 20 3393–3405. 10.1111/1462-2920.14379 30117257

[B102] Werren J. H. (2011). Selfish genetic elements, genetic conflict, and evolutionary innovation. *Proc. Natl. Acad. Sci. U.S.A.* 108 10863–10870. 10.1073/pnas.1102343108 21690392 PMC3131821

[B103] Wu B. Hao W. (2014). Horizontal transfer and gene conversion as an important driving force in shaping the landscape of mitochondrial introns. *G3* 4 605–612. 10.1534/g3.113.009910 24515269 PMC4059233

[B104] Xavier B. B. Miao V. P. Jónsson Z. O. Andrésson ÓS. (2012). Mitochondrial genomes from the lichenized fungi *Peltigera membranacea* and *Peltigera malacea*: features and phylogeny. *Fungal Biol.* 116 802–814. 10.1016/j.funbio.2012.04.013 22749167

[B105] Yang J. Zimmerly S. Perlman P. S. Lambowitz A. M. (1996). Efficient integration of an intron RNA into double-stranded DNA by reverse splicing. *Nature* 38 332–335. 10.1038/381332a0 8692273

[B106] Zeng Q. Bonocora R. P. Shub D. A. (2009). A free-standing homing endonuclease targets an intron insertion site in the psbA gene of cyanophages. *Curr. Biol.* 19 218–222. 10.1016/j.cub.2008.11.069 19200728

[B107] Zhang Y. Zhang S. Zhang G. Liu X. Wang C. Xu J. (2015). Comparison of mitochondrial genomes provides insights into intron dynamics and evolution in the caterpillar fungus Cordyceps militaris. *Fungal Genet. Biol.* 77 95–107. 10.1016/j.fgb.2015.04.009 25896956

[B108] Zimmerly S. Guo H. Eskes R. Yang J. Perlman P. S. Lambowitz A. M. (1995a). A group II intron RNA is a catalytic component of a DNA endonuclease involved in intron mobility. *Cell* 83 529–538. 10.1016/0092-8674(95)90092-6 7585955

[B109] Zimmerly S. Guo H. Perlman P. S. Lambowitz A. M. (1995b). Group II intron mobility occurs by target DNA-primed reverse transcription. *Cell* 82 545–554. 10.1016/0092-8674(95)90027-6 7664334

[B110] Zimmerly S. Hausner G. Wu X. C. (2001). Phylogenetic relationships among group II intron ORFs. *Nucleic Acids Res.* 29 1238–1250. 10.1093/nar/29.5.1238 11222775 PMC29734

[B111] Zubaer A. Wai A. Hausner G. (2018). The mitochondrial genome of Endoconidiophora resinifera is intron rich. *Sci. Rep.* 8: 17591. 10.1038/s41598-018-35926-y PMC6279837 30514960

[B112] Zubaer A. Wai A. Patel N. Perillo J. Hausner G. (2021). The mitogenomes of *Ophiostoma minus* and *Ophiostoma piliferum* and comparisons with other members of the Ophiostomatales. *Front. Microbiol.* 12:618649. 10.3389/fmicb.2021.618649 33643245 PMC7902536

