# Supplementary material for: Recent and Ongoing Horizontal Transfer of Mitochondrial Introns Between Two Fungal Tree Pathogens
Source: Front Microbiol. 2021 Jun 2;12:656609. doi: 10.3389/fmicb.2021.656609 (PMC8208691; doi:10.3389/fmicb.2021.656609)
Supplement: Supplementary file 1 [file Data_Sheet_1.PDF]

TreeBASE reviewer links for the three phylogenetic datasets are found below. These links are intended for review purposes only and should not be included in a final published version of the manuscript.

Nuclear mating type genes

http://purl.org/phylo/treebase/phylows/study/TB2:S27636?x-access-code=bac297ebaf07847a21900bda42697210&format=html

Mitochondrial alignment, all genes except *cob*

http://purl.org/phylo/treebase/phylows/study/TB2:S27637?x-access-code=aabd95c7d3ea8fcf6ff2990d8bfba0ac&format=html

Mitochondrial alignment, *cob* exons only

http://purl.org/phylo/treebase/phylows/study/TB2:S27638?x-access-code=9c42aaa9c529decd2e421f04ef168d81&format=html

The Geneious workflow mentioned in the text is available here.

https://github.com/cgmayers/QuickAssemble
