# Supplementary material for: Recent and Ongoing Horizontal Transfer of Mitochondrial Introns Between Two Fungal Tree Pathogens
Source: Front Microbiol. 2021 Jun 2;12:656609. doi: 10.3389/fmicb.2021.656609 (PMC8208691; doi:10.3389/fmicb.2021.656609)
Supplement: Supplementary file 2 [file Data_Sheet_2.docx]

**Geneious *de novo* assembly workflow**

The initial assemblies, which identified and then separately assembled low and high-coverage read pools for each isolate, were created using an automated *de novo* workflow created in Geneious comprising the following steps:

1. A random selection of 500,000 paired-end reads from the original trimmed read pool were assembled using ‘De Novo Assemble’ with default settings for ‘Low Sensitivity/Fastest’;

2. The largest 1000 contigs of this preliminary assembly were analyzed using ‘Find Low/High Coverage’ to annotate regions with ≥ 15× read coverage;

3. All regions so annotated were extracted as individual DNA sequences using the ‘Extract Annotations’ function to create the ‘high coverage filter’;

4. All reads in the original trimmed read pool were assembled using ‘Map to Reference’ with the high coverage filter as reference;

5. All reads that assembled to the high coverage filter were *de novo* assembled with default settings for ‘Medium-Low Sensitivity/Fast’ to produce the ‘high coverage assembly’;

6. All reads that did not assemble to the high coverage filter were assembled, as in step 5, to produce the ‘low coverage assembly’;

7. Consensus sequences for all contigs in the high and low coverage assemblies were generated using the ‘Highest Quality’ threshold.

Separating the high- and low-coverage read pools significantly reduced the memory required for assembly. This workflow allowed ~30 megabase fungal genomes to be assembled in less than 24 hours on a consumer PC (Windows 8, 16 GB RAM).

The full workflow, as written in Geneious, is as follows:

<geneiousWorkflows>

<XMLSerialisableRootElement name="Ceratocystis de facto de novo assembly pipeline Feb 2018 (Ver. 11.1.5 compatible)" author="" geneiousVersion="2020.1.1" uniqueId="2cf79036-c312-4235-a435-6f4e85d1a82a" revisionNumber="38" description="">

<workflowElement type="com.biomatters.plugins.workflows.WorkflowElementForEach" />

<workflowElement type="com.biomatters.plugins.workflows.WorkflowElementCustomCode">

<code>//Randomly sample 500k reads from the document to use for initial assembly. Modified from the code included in Geneious R11.0.4

import jebl.util.CompositeProgressListener;

public static List&lt;AnnotatedPluginDocument&gt; performOperation(List&lt;AnnotatedPluginDocument&gt; documents, Options options,

ProgressListener progressListener) throws DocumentOperationException {

List&lt;AnnotatedPluginDocument&gt; results = new ArrayList&lt;AnnotatedPluginDocument&gt;();

int sampleSize = 500000;

int samples = 1;

int seed = new Random().nextInt();

boolean useFirstSequences = false;

CompositeProgressListener compositeProgressListener = new CompositeProgressListener(progressListener, documents.size() * samples);

Random r = new Random(seed);

for (AnnotatedPluginDocument document : documents) {

for (int sampleSet = 1; sampleSet &lt;= samples; sampleSet++) {

compositeProgressListener.beginSubtask();

if (compositeProgressListener.isCanceled())

throw new DocumentOperationException.Canceled();

final PluginDocument pluginDocument = document.getDocument();

if (!(pluginDocument instanceof SequenceListDocument))

throw new DocumentOperationException("Can only randomly sample sequence lists");

PairedReads pairedReads = null;

List&lt;? extends SequenceDocument&gt; sequences = ((SequenceListDocument) pluginDocument).getNucleotideSequences();

SequenceListOnDisk.Builder builder;

if (sequences.isEmpty()) {

sequences = ((SequenceListDocument) pluginDocument).getAminoAcidSequences();

builder = new SequenceListOnDisk.Builder(false, SequenceDocument.Alphabet.PROTEIN, false);

} else {

builder = new SequenceListOnDisk.Builder(false, SequenceDocument.Alphabet.NUCLEOTIDE, false);

if (pluginDocument instanceof PairedReads) {

pairedReads = (PairedReads) pluginDocument;

}

}

int sequenceCount = sequences.size();

int thisSampleSize = Math.min(sequenceCount, sampleSize);

BitSet sequencesToUse = new BitSet(sequenceCount);

int sequencesPicked = 0;

while (sequencesPicked &lt; thisSampleSize) {

int sequenceIndex = useFirstSequences?sequencesPicked:r.nextInt(sequenceCount);

if (sequencesToUse.get(sequenceIndex))

continue;

sequencesToUse.set(sequenceIndex);

sequencesPicked++;

if (sequencesPicked &lt; thisSampleSize &amp;&amp; pairedReads != null &amp;&amp; pairedReads.getMateIndex(sequenceIndex) &gt;= 0) {

final int mateIndex = pairedReads.getMateIndex(sequenceIndex);

if (!sequencesToUse.get(mateIndex)) {

sequencesToUse.set(mateIndex);

sequencesPicked++;

}

}

}

sequencesPicked = 0;

for (int i = 0; i &lt; sequenceCount; i++) {

if (compositeProgressListener.setProgress(i, sequenceCount))

throw new DocumentOperationException.Canceled();

if (!sequencesToUse.get(i))

continue;

if (pairedReads != null &amp;&amp; pairedReads.getMateIndex(i) &gt;= 0 &amp;&amp; sequencesToUse.get(pairedReads.getMateIndex(i))) {

final int mateIndex = pairedReads.getMateIndex(i);

if (mateIndex &lt; i)

continue; // already done

builder.addSequenceWithMate(sequences.get(i), sequences.get(mateIndex), pairedReads.getMateExpectedDistance(i), pairedReads.getMateExpectedDistance(mateIndex), ProgressListener.EMPTY);

sequencesPicked+=2;

} else {

builder.addSequence(sequences.get(i), ProgressListener.EMPTY);

sequencesPicked++;

}

if (sequencesPicked&gt;=thisSampleSize)

break;

}

final DefaultSequenceListDocument resultDocument = builder.toSequenceListDocument(ProgressListener.EMPTY);

String name = document.getName() + " 500k";

if (samples&gt;1)

name+=" set "+sampleSet;

resultDocument.setName(name);

results.add(DocumentUtilities.createAnnotatedPluginDocument(resultDocument));

}

}

return results;

}</code>

</workflowElement>

<workflowElement id="com.biomatters.plugins.alignment.AssemblyOperation_Denovo" exposeNoOptions="true" exposeAllOptions="false" suppressErrors="false" showButtonForExposedGroup="false" groupNameForExposedOptions="" type="com.biomatters.plugins.workflows.DocumentOperationWorkflowElement">

<Options>

<option name="assemblerId">Geneious.deNovo</option>

<option name="assemblerVersion">10.2.3</option>

<option name="assemblyNamePrefixOrFolder">500k initial de novo</option>

<childOption name="data">

<option name="reassemble">true</option>

<option name="useReferenceSequence">false</option>

<option name="customComponent1" />

<option name="groupAssemblies">false</option>

<option name="namePart">0</option>

<option name="nameSeparator" extra="">- (Hyphen)</option>

<option name="customComponent2" />

<option name="assembleListsSeparately">false</option>

<option name="usePartialData">true</option>

<option name="partialDataPercentageNew">100.0</option>

</childOption>

<childOption name="method">

<childOption name="algorithm">

<option name="deNovoAssembly">Geneious.deNovo</option>

<option name="customComponent1" />

<option name="referenceAssembly">dummy</option>

<option name="customComponent2" />

</childOption>

<childOption name="biomatters.spades">

<option name="dnaSource">multiCell</option>

<option name="customComponent1" />

<option name="method">errorCorrectAndAssemble</option>

<option name="careful">true</option>

<option name="trustedContigs" />

<option name="customComponent2" />

<option name="untrustedContigs" />

<option name="customComponent3" />

<option name="overrideKmer">false</option>

<option name="kmers">21,33,55</option>

<option name="overrideThreads">false</option>

<option name="numberOfThreads">4</option>

<option name="maxMemory">14</option>

<option name="additionalOptions" />

<option name="customComponent4" />

</childOption>

<childOption name="Geneious.deNovo">

<option name="sensitivity">low</option>

<option name="customComponent1" />

<option name="fineTune">iterate_5</option>

<option name="customComponent2" />

<option name="memoryVsSpeed">5</option>

<option name="expansionWordLength">24</option>

<option name="indexWordLength">14</option>

<option name="filterRepeatsdeNovo">true</option>

<option name="filterRepeatsSizedeNovo">100</option>

<option name="allowGaps">true</option>

<option name="maxGapsPerRead">10</option>

<option name="maxGapSize">1</option>

<option name="maxMismatches">10</option>

<option name="maxAmbiguity">4</option>

<option name="applyMinOverlap">false</option>

<option name="minOverlap">25</option>

<option name="applyMinOverlapPercentageIdentical">false</option>

<option name="minOverlapPercentageIdentical">80</option>

<option name="doMoreThoroughSearching">false</option>

<option name="multipleBestMatches">mapRandomly</option>

<option name="applyMinimumMappingQuality">false</option>

<option name="minimumMappingQuality">30</option>

<option name="reanalyzeSequencesThreshold">16</option>

<option name="onlyUsePairedHitsDeNovo">false</option>

<option name="onlyMapPairedHitsReference">false</option>

<option name="onlyMapPairedHitsReferenceCombobox">mapNearby</option>

<option name="dontMergeVariantContigs">false</option>

<option name="dontMergeVariantContigsMaxCoverage">6</option>

<option name="mergeHomopolymerVariants">true</option>

<option name="allowCircularContigs">true</option>

<option name="minimumSequencesToCircularizeContig">3</option>

<option name="produceScaffolds">true</option>

<option name="linkShortOverlaps">true</option>

</childOption>

<childOption name="Tadpole">

<option name="kmer">31</option>

<option name="customComponent1" />

<option name="doPairedReadOverlap">false</option>

<option name="pairedReadOverlap">mergePairedReads</option>

<option name="minimumContigLength">200</option>

<option name="minimumCoverage">1</option>

<option name="minimumExtension">1</option>

<option name="memoryToAllocate">14000</option>

<option name="additionalOptions" />

<option name="customComponent2" />

</childOption>

<childOption name="VelvetAssemblyAlgorithm">

<option name="method">manualVelvetOptions</option>

<option name="useMultithreading">false</option>

<childOption name="manualVelvetOptions">

<option name="divider_1" />

<option name="hashLength">67</option>

<option name="isStrandSpecific">false</option>

<option name="divider_2" />

<option name="useCovCutoff">false</option>

<option name="useAutoCovCutoff">true</option>

<option name="covCutoff">0.0</option>

<option name="useMaxCovCutoff">false</option>

<option name="maxCovCutoff">200.0</option>

<option name="useExpCov">false</option>

<option name="expCov">1.0</option>

<option name="minContigLength">134</option>

<option name="allowScaffolding">true</option>

</childOption>

<childOption name="optVelvetOptions">

<option name="customComponent1" />

<option name="optMinHashLength">63</option>

<option name="optMaxHashLength">71</option>

<option name="helpButton" />

<option name="divider_1" />

<option name="optFuncKmer">n50</option>

<option name="optFuncCov">Lbp</option>

</childOption>

</childOption>

</childOption>

<childOption name="trimOptions">

<option name="method">noTrim</option>

<option name="trimOptionsButton">Options</option>

<option name="customComponent1" />

<childOption name="trimOptions">

<option name="action">annotate</option>

<childOption name="vectorTrim">

<option name="vectorScreening">false</option>

<option name="minHit">16</option>

<multiOption name="vectorDatabaseOption">

<value>

<option name="vectorDatabaseOption">UniVec</option>

</value>

</multiOption>

</childOption>

<childOption name="primerTrim">

<option name="primerScreening">false</option>

<childOption name="primerTrim">

<option name="primers" />

<option name="allowMismatches">true</option>

<option name="maxMismatches">5</option>

<option name="minLength">5</option>

</childOption>

</childOption>

<childOption name="errorProbability">

<option name="errorProbability">true</option>

<option name="errorLimit">0.05</option>

</childOption>

<childOption name="lowQualityTrim">

<option name="lowQuality">false</option>

<option name="lowQualityLimit">0</option>

</childOption>

<childOption name="ambiguityTrim">

<option name="ambiguity">false</option>

<option name="ambiguityLimit">2</option>

</childOption>

<childOption name="trimStart">

<option name="startTrim">true</option>

<option name="startTrimAtLeast">false</option>

<option name="startTrimMinimum">0</option>

</childOption>

<childOption name="trimEnd">

<option name="endTrim">true</option>

<option name="endTrimAtLeast">false</option>

<option name="endTrimMinimum">0</option>

</childOption>

<childOption name="maxLength">

<option name="use">false</option>

<option name="maxLength">1000</option>

</childOption>

</childOption>

</childOption>

<childOption name="results">

<option name="nameDeNovo">500k initial de novo</option>

<option name="addNameVariableDeNovo">...</option>

<option name="nameReference">{Reads Name} assembled to {Reference Name}</option>

<option name="addNameVariableReference">...</option>

<option name="saveReport">true</option>

<option name="customComponent1" />

<option name="saveUnusedReads">true</option>

<option name="customComponent2" />

<option name="resultsInSubfolder">true</option>

<option name="customComponent3" />

<option name="generateContigs">true</option>

<option name="limitGeneratedContigs">true</option>

<option name="generatedContigsLimit">1000</option>

<option name="customComponent4" />

<option name="generateConsensusSequencesReference">false</option>

<option name="consensusOptionsButtonReference">Options</option>

<option name="customComponent5" />

<option name="customComponent6" />

<option name="generateConsensusSequencesDeNovo">true</option>

<option name="consensusOptionsButtonDeNovo">Options</option>

<option name="customComponent7" />

<option name="customComponent8" />

<childOption name="consensus">

<option name="consensusSource">generateFromContig</option>

<childOption name="consensusOptionsReference">

<option name="thresholdPercent">weighted_60</option>

<option name="customComponent1" />

<option name="thresholdPercentNoQuality">65</option>

<option name="noConsensusGaps">false</option>

<option name="mapQuality">true</option>

<option name="mapQualityMethod">mapSummed</option>

<option name="noCoverageCharacterDeNovo">unknown</option>

<option name="noCoverageCharacterReference">unknown</option>

<option name="applyLowCoverageOrQualityCall">false</option>

<option name="lowCoverageOrQualityCharacter">unknown</option>

<option name="coverageOrQuality">coverage</option>

<option name="qualityThreshold">20</option>

<option name="coverageThreshold">2</option>

<option name="splitAroundQuestionMarks">false</option>

<option name="noConsensusEndGaps">true</option>

<option name="trimToReference">false</option>

<option name="ignoreReadsMappedToMultipleLocations">false</option>

<option name="callWhenGapInBestStates" />

<option name="howToStoreSequences">AskUser</option>

</childOption>

<childOption name="consensusOptionsDeNovo">

<option name="thresholdPercent">weighted_60</option>

<option name="customComponent1" />

<option name="thresholdPercentNoQuality">65</option>

<option name="noConsensusGaps">false</option>

<option name="mapQuality">true</option>

<option name="mapQualityMethod">mapSummed</option>

<option name="noCoverageCharacterDeNovo">unknown</option>

<option name="noCoverageCharacterReference">unknown</option>

<option name="applyLowCoverageOrQualityCall">false</option>

<option name="lowCoverageOrQualityCharacter">unknown</option>

<option name="coverageOrQuality">coverage</option>

<option name="qualityThreshold">20</option>

<option name="coverageThreshold">2</option>

<option name="splitAroundQuestionMarks">false</option>

<option name="noConsensusEndGaps">true</option>

<option name="trimToReference">false</option>

<option name="ignoreReadsMappedToMultipleLocations">false</option>

<option name="callWhenGapInBestStates" />

<option name="howToStoreSequences">AskUser</option>

</childOption>

</childOption>

</childOption>

</Options>

<optionToExpose optionName="data.partialDataPercentageNew" label="Percentage of reads to use (500k/reads, x 100):" />

</workflowElement>

<workflowElement id="FilterOperation" exposeNoOptions="true" exposeAllOptions="false" suppressErrors="false" showButtonForExposedGroup="false" groupNameForExposedOptions="" type="com.biomatters.plugins.workflows.DocumentOperationWorkflowElement">

<Options>

<option name="filterWhat">eachDocument</option>

<option name="match">all</option>

<multiOption name="filter">

<value>

<option name="field">cache_name</option>

<option name="condition">contains</option>

<option name="value">Contig</option>

</value>

</multiOption>

</Options>

<optionToExpose optionName="filterWhat" label="" />

</workflowElement>

<workflowElement type="com.biomatters.plugins.workflows.WorkflowElementGroupDocuments" />

<workflowElement id="Operation_com.biomatters.plugins.coverage.CoverageAnnotationGenerator" exposeNoOptions="true" exposeAllOptions="false" suppressErrors="false" showButtonForExposedGroup="false" groupNameForExposedOptions="" type="com.biomatters.plugins.workflows.DocumentOperationWorkflowElement">

<Options>

<option name="applyToReferencedSequences">false</option>

<childOption name="findInOptions">

<option name="onlyFindInSelectedRegion">false</option>

<option name="annotationsOnly">false</option>

<option name="annotationsOnlyType">CDS</option>

<option name="cdsOnlyType">coverageType</option>

</childOption>

<childOption name="lowCoverageOptions">

<option name="findIt">false</option>

<option name="system">standardDeviationBased</option>

<option name="limit">1</option>

<option name="standardDeviations">2.0</option>

<option name="applyMergeTolerance">true</option>

<option name="mergeTolerance">2</option>

<option name="applyMergeDistance">true</option>

<option name="mergeDistance">5</option>

</childOption>

<childOption name="highCoverageOptions">

<option name="findIt">true</option>

<option name="system">numberOfSequences</option>

<option name="limit">15</option>

<option name="standardDeviations">2.0</option>

<option name="applyMergeTolerance">true</option>

<option name="mergeTolerance">5</option>

<option name="applyMergeDistance">true</option>

<option name="mergeDistance">600</option>

</childOption>

</Options>

<optionToExpose optionName="highCoverageOptions.limit" label="" />

</workflowElement>

<workflowElement type="com.biomatters.plugins.workflows.WorkflowElementSaveAndContinue">

<options>

<option name="save">true</option>

<option name="saveInSubFolder">true</option>

<option name="subFolderName">Annotated Contigs</option>

<option name="addVariable">Include Name...</option>

<option name="selectDocuments">true</option>

<option name="doWhat">continue</option>

<option name="back">2</option>

</options>

</workflowElement>

<workflowElement id="com.biomatters.plugins.extractAnnotations.ExtractAnnotationsOperation" exposeNoOptions="true" exposeAllOptions="false" suppressErrors="false" showButtonForExposedGroup="false" groupNameForExposedOptions="" type="com.biomatters.plugins.workflows.DocumentOperationWorkflowElement">

<Options>

<option name="match">all</option>

<option name="whatToExtract">annotatedRegion</option>

<option name="intersecting">include</option>

<option name="concatenate">false</option>

<option name="includeIntergenicRegionsOption">false</option>

<option name="dontMatchTruncated">false</option>

<childOption name="extractionContextOptions">

<option name="includeUpstreamBasesOption">true</option>

<option name="numUpstreamBasesOption">150</option>

<option name="includeDownstreamBasesOption">true</option>

<option name="numDownstreamBasesOption">150</option>

</childOption>

<childOption name="consensusOptions">

<option name="thresholdPercent">weighted_60</option>

<option name="customComponent1" />

<option name="noConsensusGaps">false</option>

<option name="mapQuality">true</option>

<option name="mapQualityMethod">mapSummed</option>

<option name="noCoverageCharacterDeNovo">unknown</option>

<option name="noCoverageCharacterReference">unknown</option>

<option name="applyLowCoverageOrQualityCall">false</option>

<option name="lowCoverageOrQualityCharacter">unknown</option>

<option name="coverageOrQuality">coverage</option>

<option name="qualityThreshold">20</option>

<option name="coverageThreshold">2</option>

<option name="noConsensusEndGaps">false</option>

<option name="trimToReference">false</option>

<option name="ignoreReadsMappedToMultipleLocations">false</option>

<option name="removeGaps">true</option>

<option name="appendText">false</option>

<option name="textToAppend">consensus sequence</option>

<option name="callWhenGapInBestStates" />

<option name="howToStoreSequences">AskUser</option>

</childOption>

<multiOption name="annotation">

<value>

<option name="field">Annotation type</option>

<option name="condition">is</option>

<option name="value" extra="">High</option>

</value>

</multiOption>

</Options>

<optionToExpose optionName="match" label="" />

</workflowElement>

<workflowElement type="com.biomatters.plugins.workflows.WorkflowElementGroupDocuments" />

<workflowElement id="com.biomatters.plugins.groupSequences.GroupSequencesDocumentOperation" exposeNoOptions="true" exposeAllOptions="false" suppressErrors="false" showButtonForExposedGroup="false" groupNameForExposedOptions="" type="com.biomatters.plugins.workflows.DocumentOperationWorkflowElement">

<Options>

<option name="name">High Coverage Filter</option>

</Options>

<optionToExpose optionName="name" label="" />

</workflowElement>

<workflowElement type="com.biomatters.plugins.workflows.WorkflowElementSaveAndContinue">

<options>

<option name="save">true</option>

<option name="saveInSubFolder">true</option>

<option name="subFolderName">High Coverage Filter</option>

<option name="addVariable">Include Name...</option>

<option name="selectDocuments">false</option>

<option name="doWhat">continue</option>

<option name="back">2</option>

</options>

</workflowElement>

<workflowElement type="com.biomatters.plugins.workflows.WorkflowElementCombineWithEarlierDocuments">

<options>

<option name="back">10</option>

</options>

</workflowElement>

<workflowElement id="com.biomatters.plugins.alignment.AssemblyOperation_Reference" exposeNoOptions="true" exposeAllOptions="false" suppressErrors="false" showButtonForExposedGroup="false" groupNameForExposedOptions="" type="com.biomatters.plugins.workflows.DocumentOperationWorkflowElement">

<Options>

<option name="assemblerId">Geneious.reference</option>

<option name="assemblerVersion">10.2.3</option>

<option name="assemblyNamePrefixOrFolder">&lt;font color='red'&gt;No Documents&lt;/font&gt; assembled to none </option>

<childOption name="data">

<option name="reassemble">true</option>

<option name="useReferenceSequence">true</option>

<option name="referenceSequenceName">inMemoryDocument__|urn:local:AssemblyOptions:automaticRefSeq|Automatic___Automatically choose the most likely reference sequence from the selected documents</option>

<option name="customComponent1" />

<option name="customComponent2" />

<option name="customComponent3" />

<option name="groupAssemblies">false</option>

<option name="namePart">0</option>

<option name="nameSeparator" extra="">- (Hyphen)</option>

<option name="customComponent4" />

<option name="assembleListsSeparately">false</option>

</childOption>

<childOption name="method">

<childOption name="algorithm">

<option name="deNovoAssembly">dummy</option>

<option name="customComponent1" />

<option name="referenceAssembly">Geneious.reference</option>

<option name="customComponent2" />

</childOption>

<childOption name="Geneious.reference">

<option name="sensitivity">mediumLow</option>

<option name="customComponent1" />

<option name="findStructuralVariants">false</option>

<option name="customComponent2" />

<option name="findDeletions">false</option>

<option name="maximumDeletionSize">1000</option>

<option name="fineTune">fineTuningNone</option>

<option name="customComponent3" />

<option name="customComponent4" />

<option name="expansionWordLength">18</option>

<option name="indexWordLength">13</option>

<option name="filterRepeatsReference">true</option>

<option name="filterRepeatsSizeReference">12</option>

<option name="allowGaps">true</option>

<option name="maxGapsPerRead">10</option>

<option name="maxGapSize">15</option>

<option name="maxMismatches">20</option>

<option name="maxAmbiguity">4</option>

<option name="applyMinOverlap">false</option>

<option name="minOverlap">25</option>

<option name="applyMinOverlapPercentageIdentical">false</option>

<option name="minOverlapPercentageIdentical">80</option>

<option name="doMoreThoroughSearching">false</option>

<option name="multipleBestMatches">mapRandomly</option>

<option name="applyMinimumMappingQuality">false</option>

<option name="minimumMappingQuality">30</option>

<option name="accuratelyMapReadsWithErrorsToRepeatRegions">true</option>

<option name="trimPairedOverhangs">true</option>

<option name="includeInsertionsInStructuralVariants">true</option>

<option name="minimumJunctionSupport">2</option>

<option name="reanalyzeSequencesThreshold">16</option>

<option name="onlyUsePairedHitsDeNovo">false</option>

<option name="onlyMapPairedHitsReference">false</option>

<option name="onlyMapPairedHitsReferenceCombobox">mapNearby</option>

</childOption>

<childOption name="GeneiousRNA.reference">

<option name="sensitivity">medium</option>

<option name="customComponent1" />

<option name="RNASeqMethod">spanAnnotations</option>

<option name="intronType">mRNA</option>

<option name="maximumNovelIntronSize">500000</option>

<option name="customComponent2" />

<option name="fineTune">iterate_5</option>

<option name="customComponent3" />

<option name="expansionWordLength">14</option>

<option name="indexWordLength">12</option>

<option name="filterRepeatsReference">true</option>

<option name="filterRepeatsSizeReference">20</option>

<option name="allowGaps">true</option>

<option name="maxGapsPerRead">15</option>

<option name="maxGapSize">50</option>

<option name="maxMismatches">30</option>

<option name="maxAmbiguity">4</option>

<option name="applyMinOverlap">false</option>

<option name="minOverlap">25</option>

<option name="applyMinOverlapPercentageIdentical">false</option>

<option name="minOverlapPercentageIdentical">80</option>

<option name="doMoreThoroughSearching">false</option>

<option name="multipleBestMatches">mapRandomly</option>

<option name="applyMinimumMappingQuality">false</option>

<option name="minimumMappingQuality">30</option>

<option name="accuratelyMapReadsWithErrorsToRepeatRegions">true</option>

<option name="trimPairedOverhangs">true</option>

<option name="includeInsertionsInStructuralVariants">true</option>

<option name="minimumJunctionSupport">2</option>

<option name="reanalyzeSequencesThreshold">8</option>

<option name="onlyUsePairedHitsDeNovo">false</option>

<option name="onlyMapPairedHitsReference">false</option>

<option name="onlyMapPairedHitsReferenceCombobox">mapNearby</option>

</childOption>

</childOption>

<childOption name="trimOptions">

<option name="method">noTrim</option>

<option name="trimOptionsButton">Options</option>

<option name="customComponent1" />

<childOption name="trimOptions">

<option name="action">annotate</option>

<childOption name="vectorTrim">

<option name="vectorScreening">false</option>

<option name="minHit">16</option>

<multiOption name="vectorDatabaseOption">

<value>

<option name="vectorDatabaseOption">UniVec</option>

</value>

</multiOption>

</childOption>

<childOption name="primerTrim">

<option name="primerScreening">false</option>

<childOption name="primerTrim">

<option name="primers" />

<option name="allowMismatches">true</option>

<option name="maxMismatches">5</option>

<option name="minLength">5</option>

</childOption>

</childOption>

<childOption name="errorProbability">

<option name="errorProbability">true</option>

<option name="errorLimit">0.05</option>

</childOption>

<childOption name="lowQualityTrim">

<option name="lowQuality">false</option>

<option name="lowQualityLimit">0</option>

</childOption>

<childOption name="ambiguityTrim">

<option name="ambiguity">false</option>

<option name="ambiguityLimit">2</option>

</childOption>

<childOption name="trimStart">

<option name="startTrim">true</option>

<option name="startTrimAtLeast">false</option>

<option name="startTrimMinimum">0</option>

</childOption>

<childOption name="trimEnd">

<option name="endTrim">true</option>

<option name="endTrimAtLeast">false</option>

<option name="endTrimMinimum">0</option>

</childOption>

<childOption name="maxLength">

<option name="use">false</option>

<option name="maxLength">1000</option>

</childOption>

</childOption>

</childOption>

<childOption name="results">

<option name="nameDeNovo">{Reads Name} Assembly</option>

<option name="addNameVariableDeNovo">...</option>

<option name="nameReference">{Reads Name} assembled to {Reference Name}</option>

<option name="addNameVariableReference">...</option>

<option name="saveReport">true</option>

<option name="customComponent1" />

<option name="saveUnusedReads">true</option>

<option name="customComponent2" />

<option name="saveUsedReads">true</option>

<option name="saveUsedReadsIncludeMates">false</option>

<option name="customComponent3" />

<option name="resultsInSubfolder">false</option>

<option name="customComponent4" />

<option name="generateContigs">true</option>

<option name="limitGeneratedContigs">true</option>

<option name="generatedContigsLimit">1000</option>

<option name="customComponent5" />

<option name="generateConsensusSequencesReference">false</option>

<option name="consensusOptionsButtonReference">Options</option>

<option name="customComponent6" />

<option name="customComponent7" />

<option name="generateConsensusSequencesDeNovo">false</option>

<option name="consensusOptionsButtonDeNovo">Options</option>

<option name="customComponent8" />

<option name="customComponent9" />

<childOption name="consensus">

<option name="consensusSource">generateFromContig</option>

<childOption name="consensusOptionsReference">

<option name="thresholdPercent">weighted_60</option>

<option name="customComponent1" />

<option name="thresholdPercentNoQuality">65</option>

<option name="noConsensusGaps">false</option>

<option name="mapQuality">true</option>

<option name="mapQualityMethod">mapSummed</option>

<option name="noCoverageCharacterDeNovo">unknown</option>

<option name="noCoverageCharacterReference">unknown</option>

<option name="applyLowCoverageOrQualityCall">false</option>

<option name="lowCoverageOrQualityCharacter">unknown</option>

<option name="coverageOrQuality">coverage</option>

<option name="qualityThreshold">20</option>

<option name="coverageThreshold">2</option>

<option name="splitAroundQuestionMarks">false</option>

<option name="noConsensusEndGaps">true</option>

<option name="trimToReference">false</option>

<option name="ignoreReadsMappedToMultipleLocations">false</option>

<option name="callWhenGapInBestStates" />

<option name="howToStoreSequences">AskUser</option>

</childOption>

<childOption name="consensusOptionsDeNovo">

<option name="thresholdPercent">weighted_60</option>

<option name="customComponent1" />

<option name="thresholdPercentNoQuality">65</option>

<option name="noConsensusGaps">false</option>

<option name="mapQuality">true</option>

<option name="mapQualityMethod">mapSummed</option>

<option name="noCoverageCharacterDeNovo">unknown</option>

<option name="noCoverageCharacterReference">unknown</option>

<option name="applyLowCoverageOrQualityCall">false</option>

<option name="lowCoverageOrQualityCharacter">unknown</option>

<option name="coverageOrQuality">coverage</option>

<option name="qualityThreshold">20</option>

<option name="coverageThreshold">2</option>

<option name="splitAroundQuestionMarks">false</option>

<option name="noConsensusEndGaps">true</option>

<option name="trimToReference">false</option>

<option name="ignoreReadsMappedToMultipleLocations">false</option>

<option name="callWhenGapInBestStates" />

<option name="howToStoreSequences">AskUser</option>

</childOption>

</childOption>

</childOption>

</Options>

<optionToExpose optionName="data.reassemble" label="" />

</workflowElement>

<workflowElement type="com.biomatters.plugins.workflows.WorkflowElementGroupDocuments" />

<workflowElement type="com.biomatters.plugins.workflows.WorkflowElementSaveAndContinue">

<options>

<option name="save">true</option>

<option name="saveInSubFolder">true</option>

<option name="subFolderName">Reads mapped to filter</option>

<option name="addVariable">Include Name...</option>

<option name="selectDocuments">true</option>

<option name="doWhat">continue</option>

<option name="back">2</option>

</options>

</workflowElement>

<workflowElement id="FilterOperation" exposeNoOptions="true" exposeAllOptions="false" suppressErrors="false" showButtonForExposedGroup="false" groupNameForExposedOptions="" type="com.biomatters.plugins.workflows.DocumentOperationWorkflowElement">

<Options>

<option name="filterWhat">eachDocument</option>

<option name="match">all</option>

<multiOption name="filter">

<value>

<option name="field">cache_name</option>

<option name="condition">contains</option>

<option name="value">Used</option>

</value>

<value>

<option name="field">cache_name</option>

<option name="condition">does_not_contain</option>

<option name="value">Unused</option>

</value>

</multiOption>

</Options>

<optionToExpose optionName="filterWhat" label="" />

</workflowElement>

<workflowElement type="com.biomatters.plugins.workflows.WorkflowElementGroupDocuments" />

<workflowElement id="com.biomatters.plugins.alignment.AssemblyOperation_Denovo" exposeNoOptions="true" exposeAllOptions="false" suppressErrors="false" showButtonForExposedGroup="false" groupNameForExposedOptions="" type="com.biomatters.plugins.workflows.DocumentOperationWorkflowElement">

<Options>

<option name="assemblerId">Geneious.deNovo</option>

<option name="assemblerVersion">10.2.3</option>

<option name="assemblyNamePrefixOrFolder">High Coverage Assembly</option>

<childOption name="data">

<option name="reassemble">true</option>

<option name="useReferenceSequence">false</option>

<option name="customComponent1" />

<option name="groupAssemblies">false</option>

<option name="namePart">0</option>

<option name="nameSeparator" extra="">- (Hyphen)</option>

<option name="customComponent2" />

<option name="assembleListsSeparately">false</option>

<option name="usePartialData">true</option>

<option name="partialDataPercentageNew">100.0</option>

</childOption>

<childOption name="method">

<childOption name="algorithm">

<option name="deNovoAssembly">Geneious.deNovo</option>

<option name="customComponent1" />

<option name="referenceAssembly">dummy</option>

<option name="customComponent2" />

</childOption>

<childOption name="biomatters.spades">

<option name="dnaSource">multiCell</option>

<option name="customComponent1" />

<option name="method">errorCorrectAndAssemble</option>

<option name="careful">true</option>

<option name="trustedContigs" />

<option name="customComponent2" />

<option name="untrustedContigs" />

<option name="customComponent3" />

<option name="overrideKmer">false</option>

<option name="kmers">21,33,55</option>

<option name="overrideThreads">false</option>

<option name="numberOfThreads">4</option>

<option name="maxMemory">14</option>

<option name="additionalOptions" />

<option name="customComponent4" />

</childOption>

<childOption name="Geneious.deNovo">

<option name="sensitivity">custom</option>

<option name="customComponent1" />

<option name="fineTune">iterate_5</option>

<option name="customComponent2" />

<option name="memoryVsSpeed">4</option>

<option name="expansionWordLength">18</option>

<option name="indexWordLength">13</option>

<option name="filterRepeatsdeNovo">true</option>

<option name="filterRepeatsSizedeNovo">100</option>

<option name="allowGaps">true</option>

<option name="maxGapsPerRead">10</option>

<option name="maxGapSize">2</option>

<option name="maxMismatches">20</option>

<option name="maxAmbiguity">4</option>

<option name="applyMinOverlap">false</option>

<option name="minOverlap">25</option>

<option name="applyMinOverlapPercentageIdentical">false</option>

<option name="minOverlapPercentageIdentical">80</option>

<option name="doMoreThoroughSearching">false</option>

<option name="multipleBestMatches">mapRandomly</option>

<option name="applyMinimumMappingQuality">false</option>

<option name="minimumMappingQuality">30</option>

<option name="reanalyzeSequencesThreshold">16</option>

<option name="onlyUsePairedHitsDeNovo">false</option>

<option name="onlyMapPairedHitsReference">false</option>

<option name="onlyMapPairedHitsReferenceCombobox">mapNearby</option>

<option name="dontMergeVariantContigs">false</option>

<option name="dontMergeVariantContigsMaxCoverage">6</option>

<option name="mergeHomopolymerVariants">true</option>

<option name="allowCircularContigs">true</option>

<option name="minimumSequencesToCircularizeContig">3</option>

<option name="produceScaffolds">true</option>

<option name="linkShortOverlaps">true</option>

</childOption>

<childOption name="Tadpole">

<option name="kmer">31</option>

<option name="customComponent1" />

<option name="doPairedReadOverlap">false</option>

<option name="pairedReadOverlap">mergePairedReads</option>

<option name="minimumContigLength">200</option>

<option name="minimumCoverage">1</option>

<option name="minimumExtension">1</option>

<option name="memoryToAllocate">14000</option>

<option name="additionalOptions" />

<option name="customComponent2" />

</childOption>

<childOption name="VelvetAssemblyAlgorithm">

<option name="method">manualVelvetOptions</option>

<option name="useMultithreading">false</option>

<childOption name="manualVelvetOptions">

<option name="divider_1" />

<option name="hashLength">67</option>

<option name="isStrandSpecific">false</option>

<option name="divider_2" />

<option name="useCovCutoff">false</option>

<option name="useAutoCovCutoff">true</option>

<option name="covCutoff">0.0</option>

<option name="useMaxCovCutoff">false</option>

<option name="maxCovCutoff">200.0</option>

<option name="useExpCov">false</option>

<option name="expCov">1.0</option>

<option name="minContigLength">134</option>

<option name="allowScaffolding">true</option>

</childOption>

<childOption name="optVelvetOptions">

<option name="customComponent1" />

<option name="optMinHashLength">63</option>

<option name="optMaxHashLength">71</option>

<option name="helpButton" />

<option name="divider_1" />

<option name="optFuncKmer">n50</option>

<option name="optFuncCov">Lbp</option>

</childOption>

</childOption>

</childOption>

<childOption name="trimOptions">

<option name="method">noTrim</option>

<option name="trimOptionsButton">Options</option>

<option name="customComponent1" />

<childOption name="trimOptions">

<option name="action">annotate</option>

<childOption name="vectorTrim">

<option name="vectorScreening">false</option>

<option name="minHit">16</option>

<multiOption name="vectorDatabaseOption">

<value>

<option name="vectorDatabaseOption">UniVec</option>

</value>

</multiOption>

</childOption>

<childOption name="primerTrim">

<option name="primerScreening">false</option>

<childOption name="primerTrim">

<option name="primers" />

<option name="allowMismatches">true</option>

<option name="maxMismatches">5</option>

<option name="minLength">5</option>

</childOption>

</childOption>

<childOption name="errorProbability">

<option name="errorProbability">true</option>

<option name="errorLimit">0.05</option>

</childOption>

<childOption name="lowQualityTrim">

<option name="lowQuality">false</option>

<option name="lowQualityLimit">0</option>

</childOption>

<childOption name="ambiguityTrim">

<option name="ambiguity">false</option>

<option name="ambiguityLimit">2</option>

</childOption>

<childOption name="trimStart">

<option name="startTrim">true</option>

<option name="startTrimAtLeast">false</option>

<option name="startTrimMinimum">0</option>

</childOption>

<childOption name="trimEnd">

<option name="endTrim">true</option>

<option name="endTrimAtLeast">false</option>

<option name="endTrimMinimum">0</option>

</childOption>

<childOption name="maxLength">

<option name="use">false</option>

<option name="maxLength">1000</option>

</childOption>

</childOption>

</childOption>

<childOption name="results">

<option name="nameDeNovo">High Coverage Assembly</option>

<option name="addNameVariableDeNovo">...</option>

<option name="nameReference">{Reads Name} assembled to {Reference Name}</option>

<option name="addNameVariableReference">...</option>

<option name="saveReport">true</option>

<option name="customComponent1" />

<option name="saveUnusedReads">true</option>

<option name="customComponent2" />

<option name="resultsInSubfolder">true</option>

<option name="customComponent3" />

<option name="generateContigs">true</option>

<option name="limitGeneratedContigs">true</option>

<option name="generatedContigsLimit">1000</option>

<option name="customComponent4" />

<option name="generateConsensusSequencesReference">false</option>

<option name="consensusOptionsButtonReference">Options</option>

<option name="customComponent5" />

<option name="customComponent6" />

<option name="generateConsensusSequencesDeNovo">true</option>

<option name="consensusOptionsButtonDeNovo">Options</option>

<option name="customComponent7" />

<option name="customComponent8" />

<childOption name="consensus">

<option name="consensusSource">generateFromContig</option>

<childOption name="consensusOptionsReference">

<option name="thresholdPercent">weighted_60</option>

<option name="customComponent1" />

<option name="thresholdPercentNoQuality">65</option>

<option name="noConsensusGaps">false</option>

<option name="mapQuality">true</option>

<option name="mapQualityMethod">mapSummed</option>

<option name="noCoverageCharacterDeNovo">unknown</option>

<option name="noCoverageCharacterReference">unknown</option>

<option name="applyLowCoverageOrQualityCall">false</option>

<option name="lowCoverageOrQualityCharacter">unknown</option>

<option name="coverageOrQuality">coverage</option>

<option name="qualityThreshold">20</option>

<option name="coverageThreshold">2</option>

<option name="splitAroundQuestionMarks">false</option>

<option name="noConsensusEndGaps">true</option>

<option name="trimToReference">false</option>

<option name="ignoreReadsMappedToMultipleLocations">false</option>

<option name="callWhenGapInBestStates" />

<option name="howToStoreSequences">AskUser</option>

</childOption>

<childOption name="consensusOptionsDeNovo">

<option name="thresholdPercent">weighted_60</option>

<option name="customComponent1" />

<option name="thresholdPercentNoQuality">65</option>

<option name="noConsensusGaps">false</option>

<option name="mapQuality">true</option>

<option name="mapQualityMethod">mapSummed</option>

<option name="noCoverageCharacterDeNovo">unknown</option>

<option name="noCoverageCharacterReference">unknown</option>

<option name="applyLowCoverageOrQualityCall">false</option>

<option name="lowCoverageOrQualityCharacter">unknown</option>

<option name="coverageOrQuality">coverage</option>

<option name="qualityThreshold">20</option>

<option name="coverageThreshold">2</option>

<option name="splitAroundQuestionMarks">false</option>

<option name="noConsensusEndGaps">true</option>

<option name="trimToReference">false</option>

<option name="ignoreReadsMappedToMultipleLocations">false</option>

<option name="callWhenGapInBestStates" />

<option name="howToStoreSequences">AskUser</option>

</childOption>

</childOption>

</childOption>

</Options>

<optionToExpose optionName="data.reassemble" label="" />

</workflowElement>

<workflowElement type="com.biomatters.plugins.workflows.WorkflowElementSaveAndContinue">

<options>

<option name="save">false</option>

<option name="saveInSubFolder">false</option>

<option name="subFolderName" />

<option name="addVariable">Include Name...</option>

<option name="selectDocuments">false</option>

<option name="doWhat">branchFrom</option>

<option name="back">4</option>

</options>

</workflowElement>

<workflowElement id="FilterOperation" exposeNoOptions="true" exposeAllOptions="false" suppressErrors="false" showButtonForExposedGroup="false" groupNameForExposedOptions="" type="com.biomatters.plugins.workflows.DocumentOperationWorkflowElement">

<Options>

<option name="filterWhat">eachDocument</option>

<option name="match">all</option>

<multiOption name="filter">

<value>

<option name="field">cache_name</option>

<option name="condition">contains</option>

<option name="value">Unused</option>

</value>

</multiOption>

</Options>

<optionToExpose optionName="filterWhat" label="" />

</workflowElement>

<workflowElement type="com.biomatters.plugins.workflows.WorkflowElementGroupDocuments" />

<workflowElement id="com.biomatters.plugins.alignment.AssemblyOperation_Denovo" exposeNoOptions="true" exposeAllOptions="false" suppressErrors="true" showButtonForExposedGroup="false" groupNameForExposedOptions="" type="com.biomatters.plugins.workflows.DocumentOperationWorkflowElement">

<Options>

<option name="assemblerId">Geneious.deNovo</option>

<option name="assemblerVersion">10.2.3</option>

<option name="assemblyNamePrefixOrFolder">Low Coverage Assembly</option>

<childOption name="data">

<option name="reassemble">true</option>

<option name="useReferenceSequence">false</option>

<option name="customComponent1" />

<option name="groupAssemblies">false</option>

<option name="namePart">0</option>

<option name="nameSeparator" extra="">- (Hyphen)</option>

<option name="customComponent2" />

<option name="assembleListsSeparately">false</option>

<option name="usePartialData">true</option>

<option name="partialDataPercentageNew">100.0</option>

</childOption>

<childOption name="method">

<childOption name="algorithm">

<option name="deNovoAssembly">Geneious.deNovo</option>

<option name="customComponent1" />

<option name="referenceAssembly">dummy</option>

<option name="customComponent2" />

</childOption>

<childOption name="biomatters.spades">

<option name="dnaSource">multiCell</option>

<option name="customComponent1" />

<option name="method">errorCorrectAndAssemble</option>

<option name="careful">true</option>

<option name="trustedContigs" />

<option name="customComponent2" />

<option name="untrustedContigs" />

<option name="customComponent3" />

<option name="overrideKmer">false</option>

<option name="kmers">21,33,55</option>

<option name="overrideThreads">false</option>

<option name="numberOfThreads">4</option>

<option name="maxMemory">14</option>

<option name="additionalOptions" />

<option name="customComponent4" />

</childOption>

<childOption name="Geneious.deNovo">

<option name="sensitivity">custom</option>

<option name="customComponent1" />

<option name="fineTune">iterate_5</option>

<option name="customComponent2" />

<option name="memoryVsSpeed">4</option>

<option name="expansionWordLength">18</option>

<option name="indexWordLength">13</option>

<option name="filterRepeatsdeNovo">true</option>

<option name="filterRepeatsSizedeNovo">100</option>

<option name="allowGaps">true</option>

<option name="maxGapsPerRead">10</option>

<option name="maxGapSize">2</option>

<option name="maxMismatches">20</option>

<option name="maxAmbiguity">4</option>

<option name="applyMinOverlap">false</option>

<option name="minOverlap">25</option>

<option name="applyMinOverlapPercentageIdentical">false</option>

<option name="minOverlapPercentageIdentical">80</option>

<option name="doMoreThoroughSearching">false</option>

<option name="multipleBestMatches">mapRandomly</option>

<option name="applyMinimumMappingQuality">false</option>

<option name="minimumMappingQuality">30</option>

<option name="reanalyzeSequencesThreshold">16</option>

<option name="onlyUsePairedHitsDeNovo">false</option>

<option name="onlyMapPairedHitsReference">false</option>

<option name="onlyMapPairedHitsReferenceCombobox">mapNearby</option>

<option name="dontMergeVariantContigs">false</option>

<option name="dontMergeVariantContigsMaxCoverage">6</option>

<option name="mergeHomopolymerVariants">true</option>

<option name="allowCircularContigs">true</option>

<option name="minimumSequencesToCircularizeContig">3</option>

<option name="produceScaffolds">true</option>

<option name="linkShortOverlaps">true</option>

</childOption>

<childOption name="Tadpole">

<option name="kmer">31</option>

<option name="customComponent1" />

<option name="doPairedReadOverlap">false</option>

<option name="pairedReadOverlap">mergePairedReads</option>

<option name="minimumContigLength">200</option>

<option name="minimumCoverage">1</option>

<option name="minimumExtension">1</option>

<option name="memoryToAllocate">14000</option>

<option name="additionalOptions" />

<option name="customComponent2" />

</childOption>

<childOption name="VelvetAssemblyAlgorithm">

<option name="method">manualVelvetOptions</option>

<option name="useMultithreading">false</option>

<childOption name="manualVelvetOptions">

<option name="divider_1" />

<option name="hashLength">67</option>

<option name="isStrandSpecific">false</option>

<option name="divider_2" />

<option name="useCovCutoff">false</option>

<option name="useAutoCovCutoff">true</option>

<option name="covCutoff">0.0</option>

<option name="useMaxCovCutoff">false</option>

<option name="maxCovCutoff">200.0</option>

<option name="useExpCov">false</option>

<option name="expCov">1.0</option>

<option name="minContigLength">134</option>

<option name="allowScaffolding">true</option>

</childOption>

<childOption name="optVelvetOptions">

<option name="customComponent1" />

<option name="optMinHashLength">63</option>

<option name="optMaxHashLength">71</option>

<option name="helpButton" />

<option name="divider_1" />

<option name="optFuncKmer">n50</option>

<option name="optFuncCov">Lbp</option>

</childOption>

</childOption>

</childOption>

<childOption name="trimOptions">

<option name="method">noTrim</option>

<option name="trimOptionsButton">Options</option>

<option name="customComponent1" />

<childOption name="trimOptions">

<option name="action">annotate</option>

<childOption name="vectorTrim">

<option name="vectorScreening">false</option>

<option name="minHit">16</option>

<multiOption name="vectorDatabaseOption">

<value>

<option name="vectorDatabaseOption">UniVec</option>

</value>

</multiOption>

</childOption>

<childOption name="primerTrim">

<option name="primerScreening">false</option>

<childOption name="primerTrim">

<option name="primers" />

<option name="allowMismatches">true</option>

<option name="maxMismatches">5</option>

<option name="minLength">5</option>

</childOption>

</childOption>

<childOption name="errorProbability">

<option name="errorProbability">true</option>

<option name="errorLimit">0.05</option>

</childOption>

<childOption name="lowQualityTrim">

<option name="lowQuality">false</option>

<option name="lowQualityLimit">0</option>

</childOption>

<childOption name="ambiguityTrim">

<option name="ambiguity">false</option>

<option name="ambiguityLimit">2</option>

</childOption>

<childOption name="trimStart">

<option name="startTrim">true</option>

<option name="startTrimAtLeast">false</option>

<option name="startTrimMinimum">0</option>

</childOption>

<childOption name="trimEnd">

<option name="endTrim">true</option>

<option name="endTrimAtLeast">false</option>

<option name="endTrimMinimum">0</option>

</childOption>

<childOption name="maxLength">

<option name="use">false</option>

<option name="maxLength">1000</option>

</childOption>

</childOption>

</childOption>

<childOption name="results">

<option name="nameDeNovo">Low Coverage Assembly</option>

<option name="addNameVariableDeNovo">...</option>

<option name="nameReference">{Reads Name} assembled to {Reference Name}</option>

<option name="addNameVariableReference">...</option>

<option name="saveReport">true</option>

<option name="customComponent1" />

<option name="saveUnusedReads">true</option>

<option name="customComponent2" />

<option name="resultsInSubfolder">true</option>

<option name="customComponent3" />

<option name="generateContigs">true</option>

<option name="limitGeneratedContigs">true</option>

<option name="generatedContigsLimit">1000</option>

<option name="customComponent4" />

<option name="generateConsensusSequencesReference">false</option>

<option name="consensusOptionsButtonReference">Options</option>

<option name="customComponent5" />

<option name="customComponent6" />

<option name="generateConsensusSequencesDeNovo">true</option>

<option name="consensusOptionsButtonDeNovo">Options</option>

<option name="customComponent7" />

<option name="customComponent8" />

<childOption name="consensus">

<option name="consensusSource">generateFromContig</option>

<childOption name="consensusOptionsReference">

<option name="thresholdPercent">weighted_60</option>

<option name="customComponent1" />

<option name="thresholdPercentNoQuality">65</option>

<option name="noConsensusGaps">false</option>

<option name="mapQuality">true</option>

<option name="mapQualityMethod">mapSummed</option>

<option name="noCoverageCharacterDeNovo">unknown</option>

<option name="noCoverageCharacterReference">unknown</option>

<option name="applyLowCoverageOrQualityCall">false</option>

<option name="lowCoverageOrQualityCharacter">unknown</option>

<option name="coverageOrQuality">coverage</option>

<option name="qualityThreshold">20</option>

<option name="coverageThreshold">2</option>

<option name="splitAroundQuestionMarks">false</option>

<option name="noConsensusEndGaps">true</option>

<option name="trimToReference">false</option>

<option name="ignoreReadsMappedToMultipleLocations">false</option>

<option name="callWhenGapInBestStates" />

<option name="howToStoreSequences">AskUser</option>

</childOption>

<childOption name="consensusOptionsDeNovo">

<option name="thresholdPercent">weighted_60</option>

<option name="customComponent1" />

<option name="thresholdPercentNoQuality">65</option>

<option name="noConsensusGaps">false</option>

<option name="mapQuality">true</option>

<option name="mapQualityMethod">mapSummed</option>

<option name="noCoverageCharacterDeNovo">unknown</option>

<option name="noCoverageCharacterReference">unknown</option>

<option name="applyLowCoverageOrQualityCall">false</option>

<option name="lowCoverageOrQualityCharacter">unknown</option>

<option name="coverageOrQuality">coverage</option>

<option name="qualityThreshold">20</option>

<option name="coverageThreshold">2</option>

<option name="splitAroundQuestionMarks">false</option>

<option name="noConsensusEndGaps">true</option>

<option name="trimToReference">false</option>

<option name="ignoreReadsMappedToMultipleLocations">false</option>

<option name="callWhenGapInBestStates" />

<option name="howToStoreSequences">AskUser</option>

</childOption>

</childOption>

</childOption>

</Options>

<optionToExpose optionName="data.reassemble" label="" />

</workflowElement>

<icon>iVBORw0KGgoAAAANSUhEUgAAABgAAAAYCAYAAADgdz34AAABR0lEQVR4XtWSP0vEQBTEB0Hs5BrhOjkQa8EPoL3gn0/glZbXWQt+ChstbQQ7Sws7EQ4srhUOET2LcIWdsDfvksgy2WRjTHEOGQjZ9377drLAIsgBl2b9/mcR2qGHtDNPgWutaSwCt+gkh7e6CUF9Bed+Ak5Z0tWe2nJp3gXwNzC9APZZskmval9UTvL2/QWM9oBtlm3QK9oblSvJ2zwBbpBOvU4vSWtcLp63wYOZJzz1JzDgEEN71/W5CDpWcFXeBiKwT/DtJD3h3I/AGZeX/doffQAnObwsb9YcEnTlQ32/Anwqfr5tonlzyt0Mmigw5DvgSLAF2QTdLNcXBYQ85okfgPMBh2FvT3hhvQMHCqqA2okNvIbfXGGC7luH+mJMO2/Ac6vQgOzatQ4taMSImlg5pdLGukYaa1zaWNdYmA0yWXET/3PNAH4wVe4PD2+VAAAAAElFTkSuQmCC</icon>

</XMLSerialisableRootElement>

</geneiousWorkflows>
